# GLP1R Attenuates Sympathetic Response to High Glucose via Carotid Body Inhibition

**DOI:** 10.1161/CIRCRESAHA.121.319874

**Published:** 2022-02-01

**Authors:** Audrys G. Pauza, Pratik Thakkar, Tatjana Tasic, Igor Felippe, Paul Bishop, Michael P. Greenwood, Kristina Rysevaite-Kyguoliene, Julia Ast, Johannes Broichhagen, David J. Hodson, Helio C. Salgado, Dainius H. Pauza, Nina Japundzic-Zigon, Julian F.R. Paton, David Murphy

**Affiliations:** Bristol Medical School, Translational Health Sciences, University of Bristol, United Kingdom (A.G.P., P.B., M.P.G., D.M.).; Manaaki Manawa – The Centre for Heart Research, Department of Physiology, Faculty of Medical & Health Sciences, University of Auckland, New Zealand (P.T., I.F., J.F.R.P.).; School of Dental Medicine, University of Belgrade, Serbia (T.T.).; Institute of Anatomy, Faculty of Medicine, Lithuanian University of Health Sciences, Kaunas (K.R.-K., D.H.P.).; Institute of Metabolism and Systems Research (IMSR), and Centre of Membrane Proteins and Receptors (COMPARE), University of Birmingham, United Kingdom (J.A., D.J.H.).; Centre for Endocrinology, Diabetes and Metabolism, Birmingham Health Partners, United Kingdom (J.A., D.J.H.).; Leibniz-Forschungsinstitut für Molekulare Pharmakologie, Berlin, Germany (J.B.).; Department of Physiology, Ribeirão Preto Medical School, University of São Paulo, Brazil (H.C.S.).; Institute of Pharmacology, Clinical Pharmacology and Toxicology, Faculty of Medicine, University of Belgrade, Serbia (N.J.-Z.).

**Keywords:** carotid body, hypertension, insulin, obesity, risk factors

## Abstract

Aberrant sympathetic nerve activity exacerbates cardiovascular risk in hypertension and diabetes, which are common comorbidities, yet clinically sympathetic nerve activity remains poorly controlled. The hypertensive diabetic state is associated with increased reflex sensitivity and tonic drive from the peripheral chemoreceptors, the cause of which is unknown. We have previously shown hypertension to be critically dependent on the carotid body (CB) input in spontaneously hypertensive rat, a model that also exhibits a number of diabetic traits. CB overstimulation by insulin and leptin has been similarly implicated in the development of increased sympathetic nerve activity in metabolic syndrome and obesity. Thus, we hypothesized that in hypertensive diabetic state (spontaneously hypertensive rat), the CB is sensitized by altered metabolic signaling causing excessive sympathetic activity levels and dysfunctional reflex regulation.

**Methods:**

Using a hypothesis-free RNA-seq approach, we investigated potential molecular targets implicated in energy metabolism mediating CB sensitization and its regulation of sympathetic outflow in experimental hypertension. Identified targets were characterized using molecular and functional techniques assessing peripheral chemoreflex sensitivity in situ and in vivo.

**Results:**

We discovered GLP1R (glucagon-like peptide-1 receptor) expression in the CBs of rat and human and showed that its decreased expression is linked to sympathetic hyperactivity in rats with cardiometabolic disease. We demonstrate GLP1R to be localized to CB chemosensory cells, while targeted administration of GLP1R agonist to the CB lowered its basal discharge and attenuated chemoreflex-evoked blood pressure and sympathetic responses. Importantly, hyperglycemia-induced peripheral chemoreflex sensitization and associated basal sympathetic overactivity were abolished by GLP1R activation in the CB suggesting a role in a homeostatic response to high blood glucose.

**Conclusions:**

We show that GLP1 (glucagon-like peptide-1) modulates the peripheral chemoreflex acting on the CB, supporting this organ as a multimodal receptor. Our findings pinpoint CBs as potential targets for ameliorating excessive sympathetic activity using GLP1R agonists in the hypertensive-diabetic condition.

## Abstract 2

Supplemental Digital Content is available in the text.

**In This Issue, see p 691**

**Meet the First Author, see p 692**

**Editorial, see p 708**

Hypertension and diabetes are major risk factors for developing cardiovascular disease^1^ and are often comorbidities. Despite established clinical protocols, <40% of patients with hypertension/diabetes achieve their treatment targets and around 40% of patients in which it is controlled remain at high cardiovascular risk.^2^ This suggests that additional mechanisms must be at play to explain the remaining cardiovascular disease risk and poor target achievement rates.

**Novelty and Significance**

**What Is known?**

- Heightened sympathetic nerve activity is hallmark of essential hypertension and Type-II diabetes
- Altered sensitivity of carotid body chemoreceptors has been implicated in hypertension and metabolic syndrome in relation to increased sympathetic tone
- GLP1R (glucagon-like peptide 1 receptor) agonists are widely used antidiabetic agents with well-known cardioprotective and antihypertensive effects, the specific mechanism of which is unknown.

**What New Information Does This Article Contribute?**

- GLP1Rs are expressed in hypoxia-sensing glomus cells in the carotid body
- Activation of GLP1Rs in the carotid body acutely suppress the peripheral chemoreflex-evoked sympathetic and arterial pressure responses
- Reduction of GLP1Rs in the carotid body is associated with increased chemoreflex-evoked sympathetic drive in a model of cardiometabolic disease
- The carotid body has emerged as a potential therapeutic target to alleviate aberrant sympathetic activity in cardiorespiratory and metabolic disease. We discovered that GLP1Rs are expressed in the carotid body. Using a clinically approved GLP1R agonist, we have shown that activation of GLP1R in the carotid body suppresses carotid body-mediated cardiovascular and sympathetic responses. Further, we show that hyperglycemic-induced chemoreflex hyperreflexia is normalized by stimulation of the carotid body using a GLP1 receptor agonist. Previously, GLP1R agonists were associated with sympatho-excitation despite eliciting antihypertensive and cardioprotective effects as demonstrated in multiple randomized clinical trials. By directly measuring sympathetic nerve activity and using targeted drug delivery to the carotid body, we show a novel gut-brain axis leading to sympathoinhibition. The significance of our findings is 2-fold—our discovery serves to partly explain the antihypertensive and cardioprotective effects of GLP1R agonist therapy and may serve as a novel therapeutic target for controlling aberrant sympathetic activity in diabetes and hypertension.

Aberrant sympathetic nerve activity (SNA) is a hallmark of multiple cardiometabolic disorders including hypertension and type 2 diabetes (T2D),^3,4^ yet no current medication for either condition lowers sympathetic discharge. Carotid bodies (CBs) are polymodal sensors mediating the peripheral chemoreflex via powerful sympathetic activation. We have shown previously that hypertension is critically dependent on the CB input driving the increased sympathetic tone in spontaneously hypertensive rats^5,6^ and is ameliorated following CB ablation.^5,7^ Similarly, in patients with treatment-resistant hypertension or systolic heart failure, CB removal lowers BP and attenuates SNA,^8,9^ demonstrating the utility of therapies targeting the CBs for the treatment of sympathetically mediated cardiovascular disease.

Alongside their classical role in cardiorespiratory control, CBs are involved in whole body energy homeostasis and glucose sensitivity.^10–12^ Both hypo- and hyperglycemia significantly enhance the hypoxic ventilatory response in healthy volunteers^13^ while CB desensitization with hyperoxia attenuates this response.^14,15^ CB chemoreceptors also respond to insulin whereas hyperinsulinemia leads to CB overactivity in a model of metabolic syndrome.^16^ CB chemosensitivity is similarly shown to be increased in prediabetic patients^17^ suggesting a causal mechanism in the pathogenesis of increased SNA in T2D.^3^ Despite ongoing debate on the exact mechanism of glucose sensing in the CBs,^18^ it appears to be multimodal and incompletely defined, thus forming the basis of the present study.

Alongside hypertension, the SH rat exhibits multiple metabolic alterations including abnormal glucose metabolism, hyperinsulinemia, and insulin resistance.^19,20^ We hypothesized that this model would thus provide a unique system to study CB sensitization and sympathetic hyperactivity in the context of hypertension and metabolic disease. After all, CB denervation prevents hypercaloric diet-induced metabolic and hemodynamic alterations by reducing SNA.^16^ Thus, we sought to investigate other molecular targets implicated in energy metabolism, beyond glucose and insulin receptors, leading to sympathetic overactivity via CB sensitization.

Leading from a high-throughput transcriptomic screen, we identified the expression of GLP1R (glucagon-like peptide-1 receptor) in the CBs and show its reduced expression linked to increased sympathetic tone in the SH rat. GLP1 (glucagon-like peptide-1) is an incretin hormone secreted by the gut and a neuropeptide synthesized by proglucagon-expressing neurones in the brain. GLP1R agonists are widely used clinically for T2D treatment and have a well-document antihypertensive and cardioprotective effect, mechanistic basis of which is lacking. We show that upon activation, GLP1R in the CB chemoreceptors inhibits SNA and suppress the arterial BP response to peripheral chemoreflex stimulation in both euglycemic and hyperglycemic conditions. Together, this identifies a novel signaling circuit in which GLP1 prevents hyperglycemia-induced peripheral chemoreflex sensitization, and associated sympathetic overactivity, by peripheral chemoreceptor inhibition in a homeostatic response to high blood glucose.

## Methods

Detailed Materials and Methods and Major Resources Table are available in the Supplemental Material.

### Data Availability

RNA-seq raw sequence files and summarized read counts are publicly available at Gene Expression Omnibus under accession number: GSE178504

## Results

### Carotid Body Sensitization in SH Rat Is Linked to Altered GPCR Signaling

**Figure 1.**
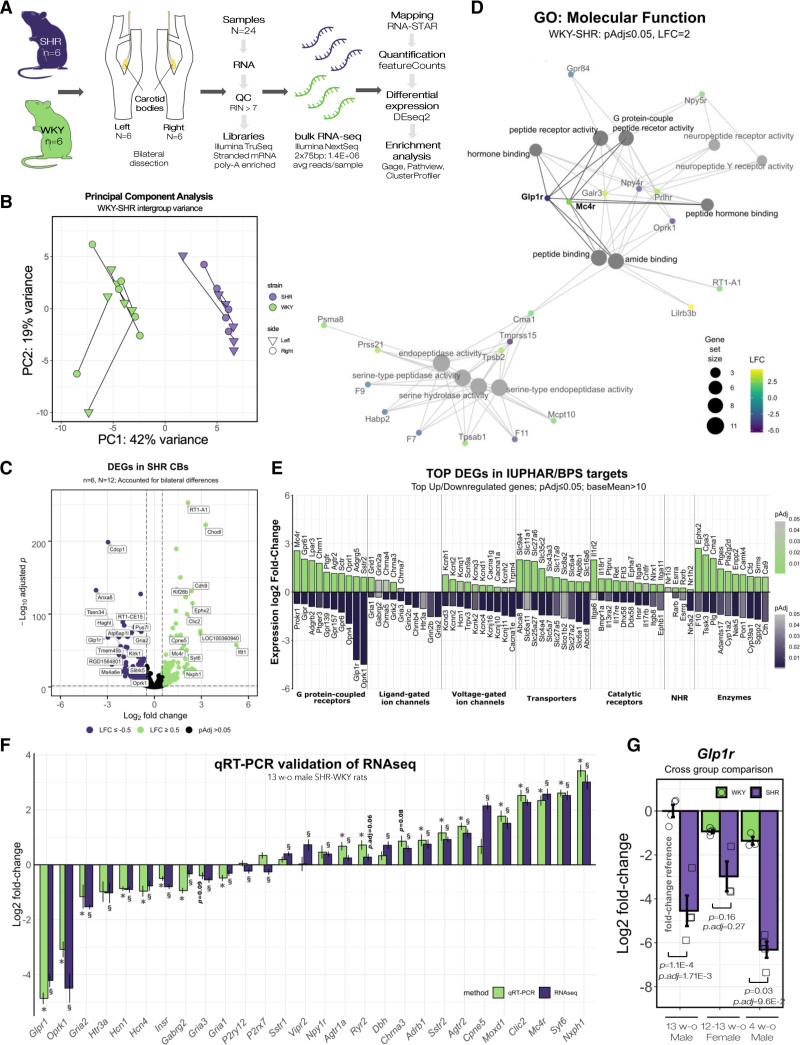
**Carotid body sensitization in SH rat is linked to altered GPCR signaling. A**, Transcriptomic study design. Bilateral carotid body (CB) samples were micro-dissected from age-matched (13 wk old) Wistar Kyoto rat (WKY)/NHsd and spontaneously hypertensive rat (SHR)/NHsd (n=6) rats. **B**, PCA plot showing distinct separation between SHR and WKY CB transcriptomes. Lines connect bilateral samples of the same animal. PC1 was attributable to strain while PC2 corresponded to sample laterality, that is, differences between sides within individuals. **C**, Volcano plot displaying differentially expressed genes (DEGs) between WKY and SHR. Dashed lines indicate LFC≥0.5 and *p*_adj_<0.05 cutoffs. **D**, Gene-concept network showing enriched GO molecular function terms. Node size and color gradient corresponds to the number and log_2_ fold-change of associated DEGs. **E**, Top 10 significantly upregulated and downregulated genes (by LFC) in SHR CBs based on IUPHAR/BPS targets. Color scale indicates *p*_adj_. Data presented in the figure are summarized in Table S9. **F**, RT-qPCR validation using an independent cohort of age-matched male WKY/NHsd and SHR/NHsd animals (n=6). Bars indicate LFC in SHR compared with WKY. Section sign (§) and asterisk (*) indicates whether expression change was significantly different in RNA-seq (^§^*p*_adj_<0.05; DEseq2, Benjamini-Hochberg correction) and RT-qPCR experiments (*P<0.05; Mann-Whitney *U*-test), respectively. Data presented in the figure is summarized in Table S10. **G**, *Glp1r* expression in male (13 wks, n=4), dioestrus female (12–13 wks, n=4), and prehypertensive male (4 wks, n=3) SHR/NHsd and WKY/NHsd CBs assessed by RT-qPCR. Data presented as relative fold-change compared with 13-wk-old Male WKY rats. Mean±SEM. Kruskal-Wallis test, Dunn post hoc test (Benjamini-Hochberg correction). NHR indicates nuclear hormone receptor.

To elucidate global gene expression changes driving the sensitized state,^6,7^ we sequenced the polyadenylated transcriptomes of bilateral CB samples from age-matched (13 weeks old) male normotensive Wistar-Kyoto (WKY/NHsd) and spontaneously hypertensive rat (NHsd) rats (Figure 1A). Principal component analysis revealed distinct separation between the SH and WKY samples (PC1=42%) with the second greatest source of variance (PC2=19%) attributable to sample laterality (ie, left versus right CB; Figure 1B). We identified a total of 2982 differentially expressed genes (DEGs, *P*.adj≥0.05, average read count across all groups (baseMean) >10) in the SH rat (Figure 1C, Data Set 1) and listed top DEGs based on IUPHAR/BPS categories highlighting potential druggable targets (Figure 1E, Table S9).

To determine affected molecular pathways associated with increased drive from the CBs, we first performed gene-ontology (GO) and pathway analysis on highly DEGs (-2≤ Log2 fold-change [LFC] ≥2).

This identified terms related to GPCR (G protein-coupled receptor) signaling to be significantly enriched in the SH rat (Data Set 2). A gene-concept network indicated changes related to peptide and amide binding to be central to CB remodeling associated with its sensitization (Figure 1D). Next, enrichment analysis was performed for an extended list of DEGs (−0.5≤LFC≥0.5) to elucidate global network-level differences linked with hypertension and altered glucose metabolism. This identified Peptide binding (GO:0042277; *P.*adj=1.78×10^−6^) and Cation channel activity (GO:0005261; *P.*adj=1.68×10^−5^) as significantly enriched GO terms. We further identified Neuroactive ligand-receptor interaction (rno04080, *P.*adj=2.0×10^−3^) and Calcium signaling (rno04020, *P.*adj=3.5×10^−2^) together with GPCR ligand binding (R-RNO-500792, *P.*adj=3.07×10^−3^) and Potassium Channels (R-RNO-1296071, *P.*adj=6.24×10^−3^) as significantly enriched KEGG and REACTOME pathways, respectively (Data Set 2).

Together, this identified multiple receptors involved in energy metabolism including Insulin receptor (*Insr*, LFC=−0.8, *P*.adj=1.69×10^−25^), glucagon-like peptide-1 receptor (*Glp1r*; LFC=−4.21, *P*.adj=1.03e-68), Gastric inhibitory polypeptide receptor (*Gipr*, LFC=-0.73, *P*.adj=1.8×10^−5^) and Melanocortin 4 receptor (*Mc4r*, LFC=2.57, *P*.adj=3.6×10^−39^) to be differentially expressed in association to CB sensitization (Figure 1C through 1E). No DEGs were detected between the bilateral samples of either strain. A full list of identified DEGs is provided in Data Set S1. Last, we validated low false discovery rate of our transcriptomic dataset in an independent cohort of age-matched male WKY and SH rats using RT-qPCR (Figure 1F; Figure S2, Table S10). In sum, high-throughput RNA profiling indicated changes related to GPCR and ion channel expression to be linked with CB sensitization driving the increased sympathetic tone in the SH rat.

### GLP1R Are Expressed in Chemosensory Glomus Cells of the Carotid Body

Leading from gene enrichment results, we sought to characterize GPCRs associated with CB sensitization (Figure 1D and 1E). GPCRs are highly druggable, thus we focused on this network to identify potential therapeutic targets. We concentrated on GLP1 receptor (*Glp1r*) as it was highly downregulated indicating a potential functional role in the enriched GPCR signaling network in the SH rat. *Glp1r* was selected based on average expression in the CBs, large expression fold-change (Figure 1E; Data Set 1) and not being previously described in the context of peripheral chemoreflex despite established links with the circulatory system and sympathetic activity.^21^ Additionally, CBs are involved in glucose homeostasis,^10,11^ the primary role of gut-derived GLP1 system, further substantiating the selection of GLP1R for further study. First, we confirmed downregulation of *Glp1r* in an independent cohort of age-matched (12-14 week) hypertensive male (Figure 1F; Figure S2A) and female SH rats (Figure 1G). This indicated there to be no statistically significant differences between sexes of either strain while being significantly reduced in SH rats compared with sex- and aged-matched WKY controls. We further found *Glp1r* to be downregulated in CBs of prehypertensive (4 weeks old) SH rats (Figure 1G) suggesting a causal rather than a consequential change in relation to the development of excessive sympathetic activity in this model.^22^

**Figure 2.**
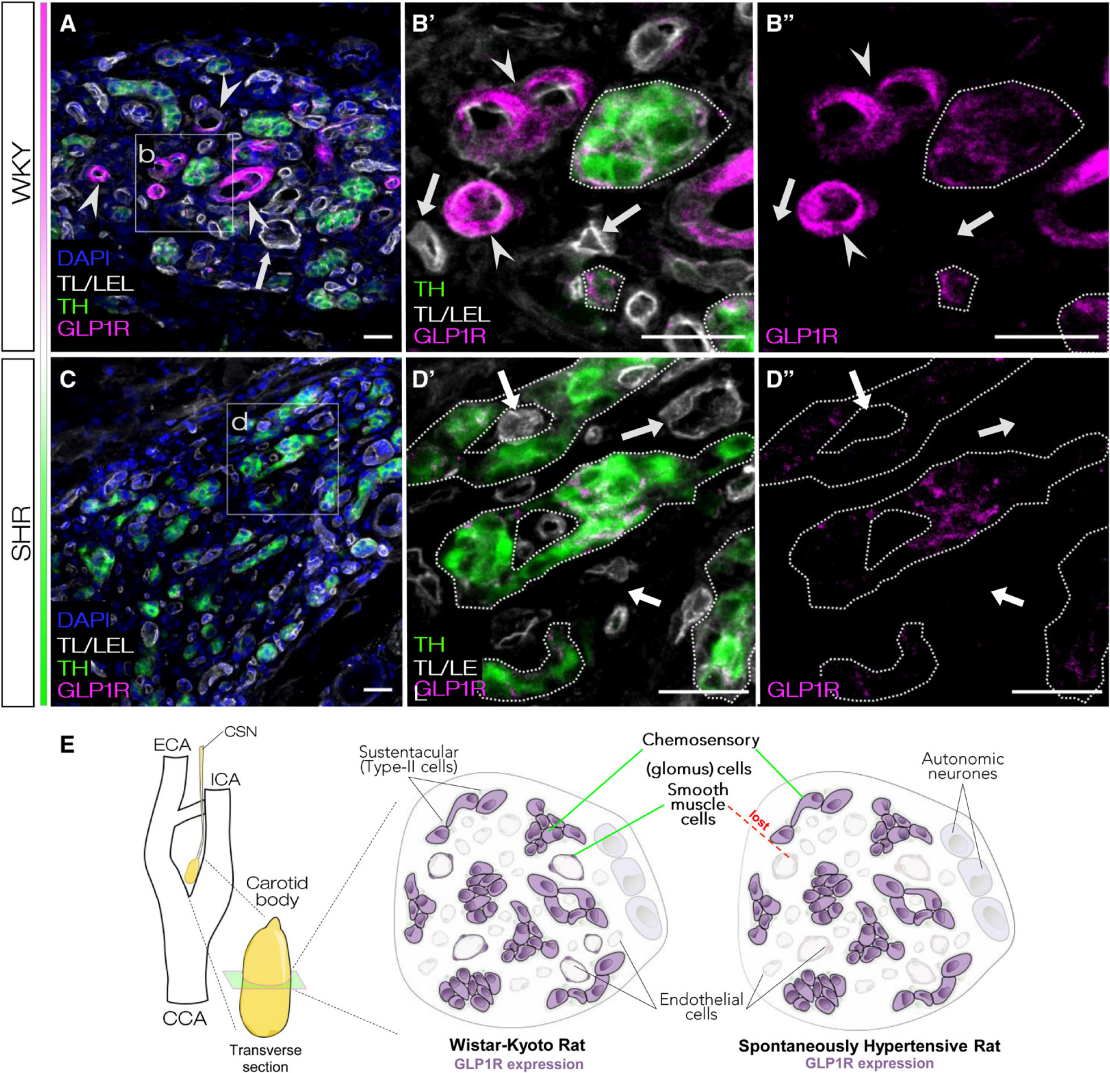
**GLP1 (glucagon like-peptide 1) receptors are expressed in chemosensory glomus cells of the carotid body. A** and **B**, GLP1R (glucagon like-peptide 1 receptor) were found to be present in the chemosensory glomus cells as shown by cross-immunoreactivity with glomus cell marker (TH). GLP1R was also expressed in a subset but not all blood vessels associated with tomato lectin/lycopersicon esculentum lectin (TL/LEL) signal in WKY carotid bodies (CBs). **C** and **D**, CBs of SH rats contained no GLP1R-immunoreactive blood vessels while expression in the chemosensory component was retained. **E**, Diagram summarizing GLP1R localization in rat CBs. In WKY, GLP1R is localized in chemosensory glomus cells and a subset but not all blood vessels. In SH rats, GLP1R is retained in the chemosensory component but is lost from the vascular component. Representative images selected based on GLP1R localization across all WKY and SHR samples (Figure S4). Magnified panels selected to highlight differences in GLP1R localization between WKY and SHR. Arrowheads—blood vessels expressing GLP1R; Arrows—blood vessels not expressing GLP1R. Images representative of n=3. Scale bar—20 µm. DAPI indicates 4′,6-diamidino-2-phenylindole; and TH, tyrosine hydroxylase.

Next, we characterized presence of GLP1R in the CBs using immunofluorescence and GLP1R antagonistic peptide label. For this, we used anti-GLP1R antibody (ab218532, Abcam), which we robustly validated for specificity in cultured cells and known sites of GLP1R expression (see Methods, Figures S3 and S5B). In WKY, GLP1R was detected in the TH-positive glomus cells and in association with a subset of endothelial cell marker (tomato lectin/lycopersicon esculentum lectin)-positive blood vessels (Figure 2A and 2B, Figure S4A). In contrast, GLP1R was localized solely to the glomus cell clusters in SH rats (Figure 2C and 2D, Figure S4B). We re-confirmed these results and showed accessibility of GLP1R antagonists to the CBs using a fluorescent GLP1R antagonistic peptide label LUXendin645.^23^ LUXendin645 labeling showed analogous pattern to the antibody staining in the CB (Figure S5). Intense labeling with LUXendin645 was localized to the chemosensory glomus cells in both strains. In contrast to normotensive WKY, no LUXendin645 labeling was associated with the Tomato lectin/lycopersicon esculentum lectin-positive vascular component in the SH rat (Figure S5A).

Together, this shows that in rat CBs GLP1R is localized to the chemosensory glomus cells and a subset of blood vessels supplying the peripheral chemoreceptors (Figure 2E). Using 2 independent approaches, we show downregulation of *Glp1r* in the SH rat to originate from the loss of GLP1R production in the CB vascular component, similar to other vascular beds in this model.^24^

### GLP1R Modulate Basal Sympathetic Drive From Peripheral Chemoreceptors

**Figure 3.**
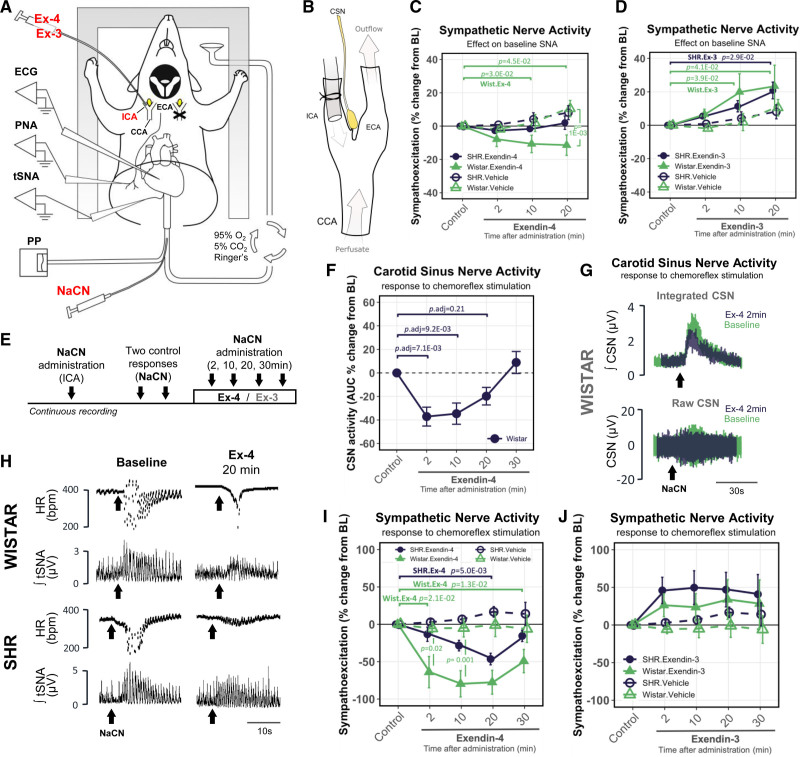
**GLP1 (glucagon-like peptide 1) receptors modulate basal and chemoreflex-evoked sympathetic nerve activity mediated by the peripheral chemoreceptors. A**, Diagram of in situ working heart-brain stem preparation (WHBP). Recordings were made unilaterally targeting the right carotid body (CB) while contralateral common carotid artery was occluded. NaCN was administered into the thoracic aorta to evoke peripheral chemoreflex response. **B**, Drugs were precisely administered to the CB via cannulated internal carotid artery (ICA). After accessing the CB, drugs were washed off via the external carotid artery (ECA) bypassing the brain. **C**, Changes in basal thoracic sympathetic nerve activity (tSNA) in response to Exendin-4 and (**D**) Exendin-3 administration in Wistar and SH rats. n=4. **E**, Experimental protocol for testing the peripheral CB chemoreceptor sensitivity over a series of chemoreflex challenges. **F**, Changes in carotid sinus nerve (CSN) activity in response to Exendin-4 administration in Wistar rats. n=6. Kruskal-Wallis test, Dunn’s post hoc test (Bonferroni correction). **G**, Raw and integrated chemoreflex-evoked CSN activity traces before and 2 min following Ex-4 administration. Representative traces selected based on similarity to average values obtained across all animals for control and 2 min timepoints. **H**, Representative traces of chemoreflex-evoked HR and integrated tSNA responses to NaCN at baseline and 20 min following Exendin-4 administration in Wistar and SH rats. Representative traces selected to best match the average values obtained across all animals. **I**, Changes in chemoreflex-evoked tSNA response following Exendin-4 (n=8) and (**J**) Exendin-3 (n=6) administration in Wistar and SH rats. Nonparametric Friedman test (within-group) and the Kruskal-Wallis test (between-group), Dunn’s post hoc test (Bonferroni correction). Arrows indicate NaCN bolus. Mean±SEM. PP indicates pulse pressure.

Following the discovery of GLP1R expression in the CBs, we tested if pharmacological activation/inhibition of this receptor would affect the peripheral chemoreflex. We recorded thoracic sympathetic nerve activity (tSNA) and carotid sinus nerve activity using an in situ working heart-brainstem preparation (WHBP) (see Methods, Figure 3A). This permitted highly targeted drug administration to the CB via the internal carotid artery (Figure 3B) where drugs were subsequently washed away via the external carotid branch without accessing the brain and activating central GLP1 receptors.

First, we determined that change in *Glp1r* expression compared with spontaneously hypertensive rat was similar in WKY and outbred Wistar rats used as controls (Figure S6B). Wistar controls were selected due to the known heterogeneity of WKY colonies around the world^25^ and being outbred served to better represent response heterogeneity within the human population. Consistent with previous reports, baseline tSNA was significantly higher in the SH compared with Wistar rats (Figure S6C).^26^ Administration of Exendin-4 (Ex-4; GLP1R agonist) to the CB reduced resting tSNA by −11.4±6.1% 20 minutes following administration in normotensive Wistar but not in the SH rat (Figure 3C, Table S2). Whereas blocking of GLP1R in the CB by use of Exendin-3 (9-39) amide (Ex-3; GLP1R antagonist) significantly increased resting tSNA by ≈20%–23% in both strains 20 minutes following administration (Figure 3D, Table S3). No significant change from baseline was detected in resting tSNA level using vehicle control in either strain.

Finding that Ex-3 potentiated resting tSNA hinted that GLP1 could be released within the CB to modulate its activity. To investigate this, we visualized biologically active GLP1 (7-36) amide in WKY and SH rats using immunofluorescence (Figure S7). GLP1-positive nerve fibers were scattered and entangled TH-positive glomus cells within the CBs. GLP1 signal was concentrated in structures resembling synaptic boutons and found in close association to GLP1R-positive chemosensory cells. No glucagon (*Gcg*) mRNA was detected in the CBs (Data set 1) indicating GLP1 to likely originate from petrosal afferents and/or superior cervical ganglion motor projections supplying the CBs.^27^ Together, these data show that GLP1R ligands suppress CB-driven sympathetic outflow and that GLP1 may be released in the CBs to modulate its activity.

### Activation of GLP1R in the Carotid Body Suppresses Chemoreflex-Evoked Sympathetic Activity

After showing that GLP1R modulate basal drive from the CBs, we tested if activation/inhibition of GLP1R would affect the peripheral chemoreceptor sensitivity. Sodium cyanide (NaCN) was used to chemically stimulate the peripheral chemoreflex as this treatment provides highly targeted, temporally controlled, and a quantifiable stimulus targeting specifically the CBs.^5,7^ Chemoreceptor sensitivity was measured as change in the magnitude of chemoreflex-evoked response to a supra-threshold, sub-maximal dose of NaCN (Figure S8) before and after drug administration. Peripheral chemoreflex was stimulated in a time-course experiment by a series of NaCN challenges after registering a pair of control responses (Figure 3E). In Wistar controls, Ex-4 significantly reduced the chemoreflex-evoked carotid sinus nerve outburst by −37.11% 2 minutes following administration (Figure 3F and 3G). Further, Ex-4 reduced the chemoreflex-evoked tSNA bursts by up to −79.6% compared with baseline response and peaked between 10 and 20 minutes after administration in Wistars (Figure 3H and 3I, Table S2). Consistent with reduced *Glp1r* expression, this effect was strongly reduced in the SH rat. Next, we applied Ex-3, which enhanced the chemoreflex-evoked tSNA response for the duration of the experiment (Figure 3J, Table S3) and did not differ between the strains. No change in chemoreflex-evoked tSNA responses were detected in both vehicle and time controls of either strain. Next, Ex-4 and Ex-3 were administered in sequence (Figure S6A). This indicated Ex-3 to overturn the Ex-4 inhibitory effect on chemoreflex-evoked tSNA response (Figure S6D, Table S4). No changes in phrenic nerve activity, respiratory rate, and pulse pressure were detected in response to Ex-4 and Ex-3 administration in either strain. Together, these data show that GLP1R activation in the CBs reduces peripheral chemoreceptor sensitivity and inhibits chemoreflex-evoked sympathetic activity selectively.

### GLP1R Agonists Attenuates Chemoreflex-Evoked Arterial Blood Pressure Response In Vivo

**Figure 4.**
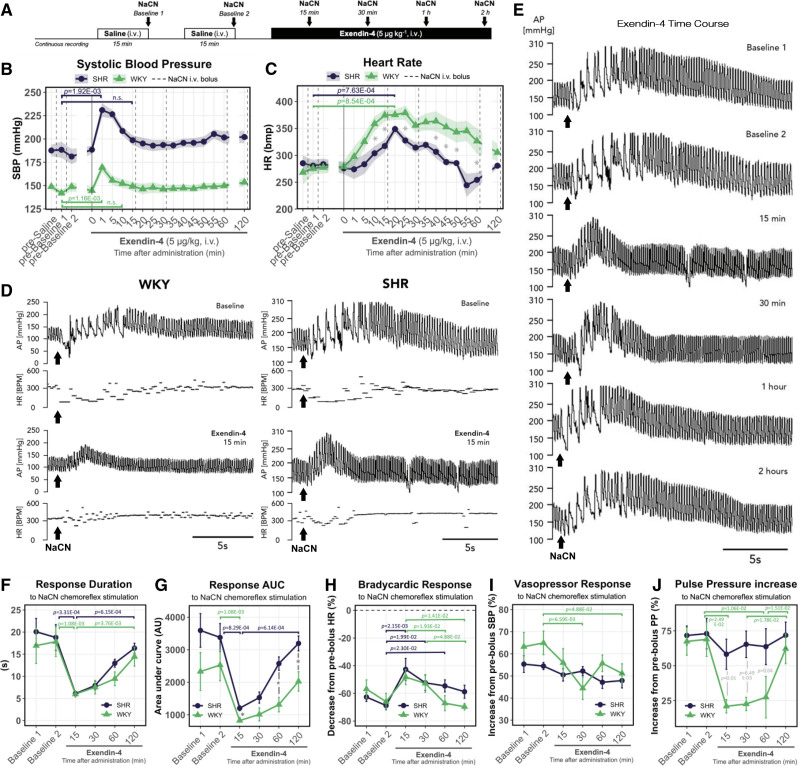
**Systemic GLP1 (glucagon-like peptide 1) receptor activation suppresses chemoreflex-evoked arterial BP response in vivo. A**, Experimental protocol for testing peripheral chemosensitivity in vivo. **B**, Resting systolic BP and (**C**) HR responses to Exendin-4 (5 µg kg^−1^, IV) administration. Dotted lines indicate chemoreflex stimulus (NaCN). Solid line indicates Exendin-4 administration. **D**, Representative traces of chemoreflex-evoked BP and HR responses before and 15 min after Exendin-4 administration in SH and WKY rats. Traces for SH and WKY are shown on different scales to highlight the chemoreflex-evoked responses in normotensive conditions. Representative traces selected to best match the average values across all animals in each group as shown in **F–J**. **E**, Representative time-course traces of chemoreflex-evoked BP responses following Exendin-4 administration. Representative traces of this specific SH rat selected to best match the average values across all animals as shown in **F** and **G**. **F**, Changes in chemoreflex-evoked BP response duration and (**G**) area under curve following Exendin-4 administration. Exendin-4 effect on chemoreflex-evoked (**H**) bradycardic, (**I**) vasopressor, and (**J**) pulse pressure (PP) increase responses. Bradycardic response (**H**) measured as the maximum drop in HR in response to chemoreflex stimulation (NaCN). Values expressed as percentage decrease compared with average HR prior the chemoreflex stimulation. Pressor response **I** measured same as **H** but for maximum increase in systolic BP. PP response **J** measured same as **H** but for maximum increase in PP during the post-peak period of the pressor response (devoid of bradycardic episodes). Kruskal-Wallis, Dunn’s post hoc test with Bonferroni correction. n=6. Mean±SEM. Arrows indicate NaCN bolus. *P<0.05.

Next, we investigated if the peripheral chemoreflex inhibition is present following a systemic activation of GLP1R more comparable to a clinical setting. For this, we again tested the peripheral chemoreflex sensitivity using a series of NaCN challenges after Ex-4 administration (5 µg kg^−1^, IV) in conscious instrumented SH and WKY rats (Figure 4A; Table S1).

GLP1R agonists have well-documented cardiovascular effects, thus we first assessed Ex-4 action on basal hemodynamic parameters (Figure S10). In line with previous reports,^28^ Ex-4 produced an acute and transient rise in resting BP that returned to baseline at first chemoreflex challenge timepoint (Figure 4B). Ex-4 also produced a delayed ramp up in resting heart rate that peaked between 15 and 30 minutes post-administration (Figure 4C), likely originating from direct sinoatrial node activation and/or decreased cardiac vagal outflow.^29,30^

Next, we assessed Ex-4 effects on the peripheral chemoreflex. Ex-4 powerfully suppressed, although not completely abolished, the chemoreflex-evoked arterial blood pressure response (Figure 4D). This effect was transient and coincided with Ex-4 elimination time reported by previous pharmacokinetic studies^31^ (Figure 4E). First, 15 minutes following Ex-4 administration the duration and area under curve of the chemoreflex-evoked BP response was reduced by >65% in both strains (Figure 4F and 4G). Ex-4 also transiently attenuated the chemoreflex-evoked vasopressor response by 30.7% 30 minutes following administration in the WKY but not in the SH rat (Figure 4I). Further, Ex-4 alleviated the chemoreflex-evoked bradycardic response in both strains (Figure 4H). This effect displayed no interstrain differences and peaked at 15 minutes after Ex-4 administration (Figure 4E). Peripheral chemoreflex stimulation induced a transient widening of pulse pressure (PP) most prominent during the post-peak period of the response (Figure 3D and 3E). Fifteen minutes after Ex-4 administration, this effect was reduced by 19.5% and 69.3% in SH and WKY strains, respectively (Figure 4J). Suppression of the PP widening was greatly reduced in the SH rat. No differences were detected between any of the baseline responses.

Last, using spectral analysis of systolic BP variability and heart rate variability we investigated whether peripheral chemoreflex suppression resulted in reduced sympathetic drive (Figure S11). Ex-4 administration reduced the sympathetic vasomotor tone as indicated by a reduction in low frequency (LF) domain of systolic BP variability (Figure S11D). Similarly, spectral analysis of heart rate variability indicated cardiac autonomic balance to be shifted toward reduced sympathetic influence as indicated by a decrease in LF:HF ratio in response to Ex-4 (Figure S11I). In stark contrast, no changes in baroreflex sensitivity were induced by Ex-4 in either strain (Figure S10H). Together, these data show that Exendin-4 acutely suppresses the peripheral chemoreceptors in vivo.

### Activation of Carotid Body GLP1 Receptors Eliminates Glucose Evoked Sympathetic Sensitization

**Figure 5.**
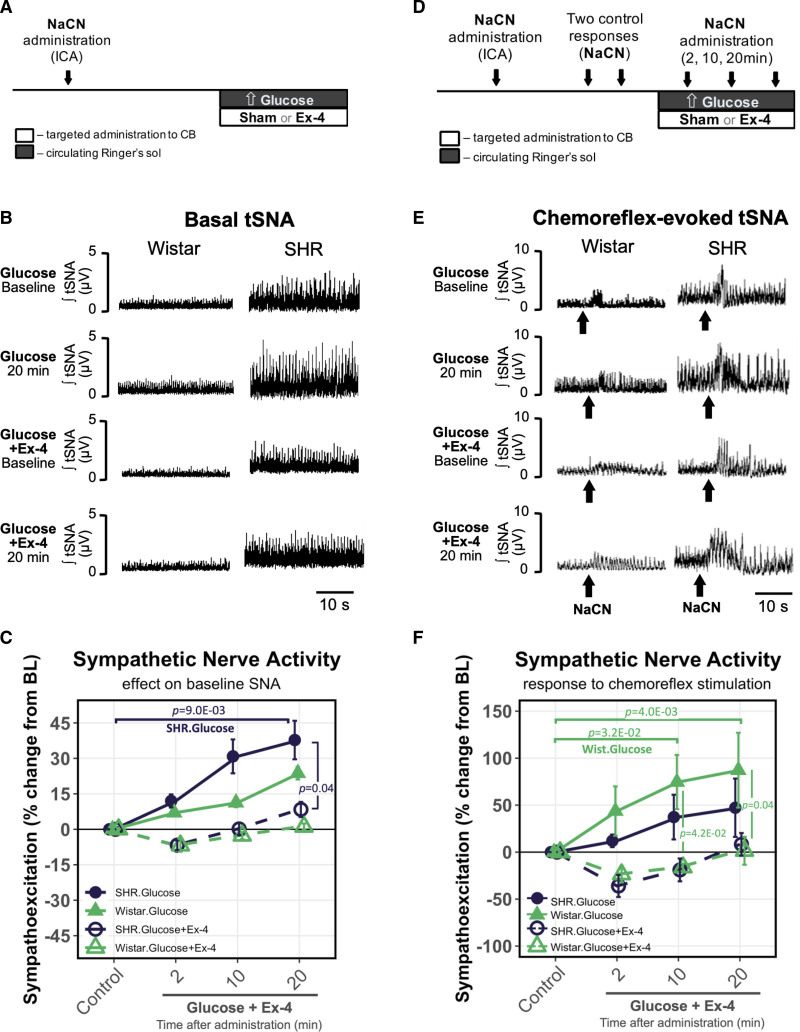
**Activation of carotid body GLP1 (glucagon-like peptide 1) receptors eliminates glucose-evoked sympathetic sensitization. A**, Experimental protocol for testing high-glucose and Ex-4 effects on basal thoracic sympathetic nerve activity (tSNA). Glucose concentration was increased in circulating Ringer’s solution from 10 to 30 mmol/L while simultaneously administering either sham or Ex-4 to the carotid body (CB) as shown in Figure 3B. **B**, Representative traces of Exendin-4 effect on basal tSNA at baseline and 20 min following hyperglycemic stimulus in Wistar and SH rats. **C**, Changes in basal tSNA in response to hyperglycemic stimulus with and without Exendin-4. High-glucose alone resulted in peripheral chemosensory sensitization that was normalized by Ex-4 administration to the CB. Wistar n=4; SHR n=5. **D**, Experimental protocol for testing high-glucose and Ex-4 effects on chemoreflex-evoked tSNA. After 2 similar control responses to NaCN, hyperglycemic stimulus and Ex-4 was administered simultaneously as detailed in **A**. **E**, Representative traces of Exendin-4 effect on chemoreflex-evoked tSNA at baseline and 20 min following hyperglycemic stimulus in Wistar and SH rats. Arrows indicate NaCN bolus. **F**, Changes in chemoreflex-evoked tSNA in response to hyperglycemic stimulus with and without Exendin-4. High glucose–induced peripheral chemoreflex sensitization was normalized by Ex-4. n=4. Representative traces shown in **B** and **E** were selected to best match the average values across all animals in each group as shown in **C** and **F**, respectively. Nonparametric Friedman test (within-group) and the Kruskal-Wallis test (between-group), Dunn’s post hoc test (Bonferroni correction). Mean±SEM.

The CBs are implicated in systemic glycemic control and respond to changes in blood sugar levels.^12,13^ To explain the physiological role of CB chemoreceptor inhibition via GLP1R, we investigate the interplay between the chemosensory GLP1R activation and sympathetic sensitization to glucose using WHBP (Figure 3A). For this, we simultaneously increased the glucose concentration in the circulating Ringer’s solution from 10 to 30 mmol L^−1^ (Figure 5A and 5D) and administered either Ex-4 or sham (saline) to the CB (Figure 3B). Increase in glucose concentration alone resulted in chemosensory sensitization as shown by an increase in basal tSNA drive by 23.7% and 37.8% in Wistar and SH rats, respectively (Figure 5B and 5C, Table S5). Increased glucose concentration also augmented chemoreflex-evoked tSNA response in Wistar (87.2%) and SH rats (47.3%) (Figure 5E and 5F). Remarkably, simultaneous administration of Ex-4 counteracted the hyperglycemia-induced peripheral chemosensory sensitization and normalized to baseline levels both resting and chemoreflex-evoked tSNA responses in both strains (Figure 5B, 5C, 5E, and 5F; Table S5).

### GLP1 Receptors Are Expressed in Human Carotid Bodies

**Figure 6.**
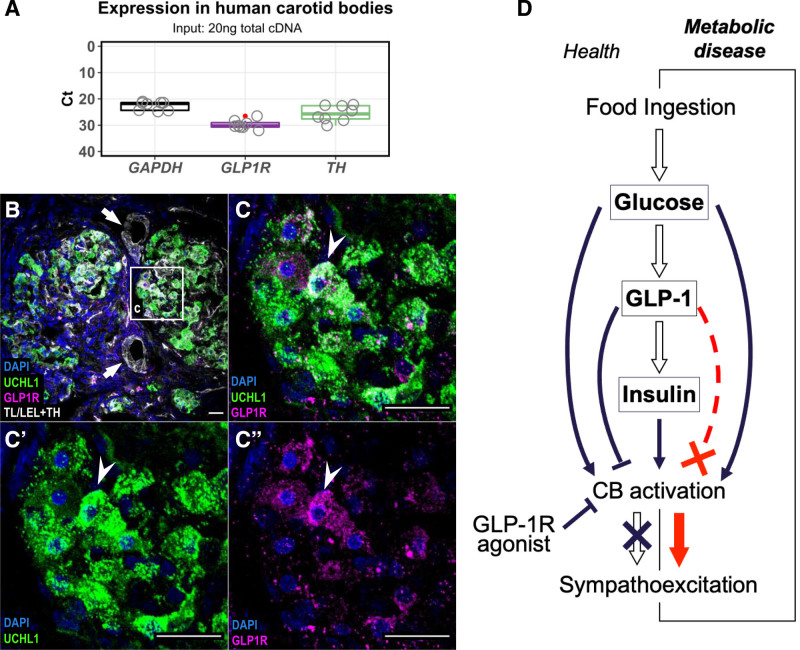
**GLP1 (glucagon-like peptide 1) receptor expression in human carotid bodies. A**, *GLP1R* mRNA expression in human carotid bodies (CBs) assessed by RT-qPCR. Expression presented as Ct values in comparison to housekeeping (*GAPDH*) and reference (*TH*) genes. All reactions were performed on the same plate. *TH* – Tyrosine Hydroxylase; Boxplot hinges represent interquartile range (IQR=Q3–Q1). Red dot indicates an outlier (Q3+1.5*IQR). n=5. **B** and **C**, Localization of GLP1R in human CBs. GLP1R immunoreactivity (magenta) was detected in chemosensory glomus cells marked by UCHL1 (green). Arrows indicate blood vessels devoid of GLP1R immunoreactivity. Arrowhead indicates GLP1R-positive chemosensory glomus cell. n=1. Representative image selected best demonstrating GLP1R localization in a well-defined chemosensory glomus cell cluster. Scale bar—20 µm. **D**, Schematic model of GLP1 action on CBs in regulating sympathetic activity. Upon food ingestions, rise in blood glucose activates the CBs leading to sympathoexcitation and stimulates release of GLP1 from intestinal L-cells. GLP1 mediates insulin secretion which additively stimulates the CB chemoreceptors. GLP1 inhibits the chemosensory CB cells counteracting the sympathoexcitation to elevated levels of glucose and/or insulin in normal physiological context. Disruption of the GLP1 inhibitory component leads to aberrant sympathoexcitation as shown in the SH model. Treatment with GLP1R agonists act to reduce sympathetic activity by suppressing the peripheral chemoreflex drive from the CBs. TL/LEL indicates tomato lectin/lycopersicon esculentum lectin; and UCHL1, ubiquitin carboxy-terminal hydrolase L1.

If the described sympathoinhibitory effect on peripheral chemoreflex is to be considered in humans, then it is essential to assess whether GLP1R expression in the CBs is conserved across species. CBs were obtained from human cadavers and GLP1R expression was determined on mRNA and protein levels. *GLP1R* mRNA was detected in all samples (n=5) and presented in relation to the housekeeping (*GAPDH*) and chemosensory cell marker (*TH*) gene expression (Figure 6A). Next, we assessed the production of GLP1R by immunohistochemistry (n=1) using previously validated anti-GLP1R (ab218532) antibody (Figure S3). We confirmed GLP1R to be localized to chemosensory glomus cells and overlap with pan-neuronal marker UCHL1 (Figure 6C). Expression pattern in humans paralleled that observed in SH rats (Figure 2) as no GLP1R immunoreactivity was observed in association to tomato lectin/lycopersicon esculentum lectin-positive vascular components. Together, these data demonstrate that GLP1R is present in human CBs with important therapeutic implications considering the use of GLP1R agonists.

## Discussion

Here, we describe GLP1R expression in the CBs and show it to be implicated in the peripheral chemoreflex sensitization in conditions of elevated BP and altered glucose metabolism. By use of selective GLP1R agonist/antagonist delivered focally to the CB, we demonstrate that both exogenous and endogenous ligands acting on GLP1R modulate tSNA via CB chemoreceptor inhibition. We further show that activation of GLP1R in the CBs attenuates its reflex sensitivity and reduces tonic afferent drive from the peripheral chemoreceptors in a homeostatic response to high glucose. Finally, we demonstrate that this inhibitory effect is present following a systemic administration of GLP1R agonists in vivo. To explain these data, we propose a novel mechanism by which in health GLP1 acts to prevent chemoreflex-mediated sympathetic activation in response to increased levels of glucose, and/or insulin, during the postprandial period, and leading to aberrant sympathetic outflow in metabolic dysfunction (Figure 6D).

Our data indicate decreased production of GLP1R in CBs to be linked to sympathetic hyperactivity in the SH rat. This is shown by a higher basal tSNA (Figure 3C) and augmented chemoreflex-evoked tSNA (Figure 3I) responses following Exendin-4 administration in SHR versus Wistar rat. Interestingly, the effects of GLP1R activation on chemoreflex-evoked responses were partly retained in the SH rats despite substantially lower *Glp1r* expression in the CBs. Further analysis revealed that in the SH rat GLP1R is lost from the media of blood vessels supplying the CBs similar to renal vasculature in this model,^24^ however, is retained in the chemosensory glomus component explaining the retained chemosensory suppression by GLP1R agonist. It is possible that loss of GLP1R in the CB vasculature contributes to enhanced chemoreflex gain as reduced CB blood flow was previously shown to augment peripheral chemosensitivity.^32^ Importantly, when using the reduced WHBP preparation, drugs were administered unilaterally into the internal carotid artery (Figure 3B) as a concentrated bolus (30 μL); this way after accessing the CB, any agents were washed away into a large volume (200 mL) of recirculating perfusate preventing activation of brain GLP1R. The WHBP is also devoid of the gastrointestinal tract, adrenal glands, kidneys, and pancreas (Figure 3A) restricting response specificity to the CB. Additive inhibitory effects of secondary messengers such as ANP (atrial natriuretic peptide),^33^ which were previously shown to be released in response to systemic GLP1R stimulation cannot be completely ruled out.^34^ However, this is unlikely due to precise drug administration and its rapid dilution in a large volume. The finding that Exendin-4 reduced the duration and overall magnitude of the chemoreflex-evoked BP response in unanesthetized, freely moving animals (Figure 4) parallels the WHBP data and demonstrates that the peripheral chemoreflex suppression is present with systemic GLP1R activation. Chemoreflex bradycardia was also attenuated by Exendin-4 (Figures 3H and 4D); however, this effect was short lived (Figure 4I) and variable using WHBP (Figure S6E). We also failed to see any modulation of the chemoreflex-evoked tachypnea. We explain this by the ribbon cable hypothesis in which subsets of glomus cells expressing GLP1R may be connected to distinct reflex pathways.^35^ Together, our data indicate that activation of GLP1R in the CBs preferentially modulates the sympathetic chemoreflex responses. This is supported by our finding that Exendin-4 reduced the basal sympathetic vasomotor tone in association to suppressing the peripheral chemosensory drive.

Conflicting data exist regarding the effects of GLP1R agonists (GLP1RAs) on sympathetic activity. Several studies have shown that acutely GLP1RAs activates the sympathetic nervous system both in experimental models^29,36–38^ and humans.^39^ However, prolonged treatment with GLP1RAs in T2D patients have an established antihypertensive effect and reduces risk of major adverse cardiovascular events^40^ making chronic sympathoexcitation unlikely. This discrepancy is thought to arise from interspecies differences; however, a definite mechanism for the antihypertensive/cardioprotective effect of GLP1RAs in humans is lacking.^41,42^ Previously, liraglutide, a long-acting GLP1RA, was shown to reduce BP via indirect action of ANP^34^; however, reports in healthy volunteers^43^ and T2D patients^44^ found no evidence of GLP1–ANP axis in humans. The antihypertensive effect of GLP1RAs also cannot be attributed to weight loss as it occurs early after the start of treatment^42^ suggesting alternative mechanism/s must be involved to explain the BP reduction. Recent report using SH rats showed 14 days treatment with liraglutide to alleviate hypertension and reduce markers of neural activation in brain regions associated with sympathetic activity, despite showing an opposite pattern of brain activation acutely.^45^ Another study in SH rats reported 28-day treatment with Exendin-4 to markedly reduce BP by 12±1 mm Hg.^46^ Considering that hypertension is critically dependent on CB input in this model,^7^ and in the light of our results, the observed BP reduction in both studies is likely to have involved a tonic inhibition of the CBs leading to reduced sympathetic drive.

The interplay between neuronal (central) and gut-derived (peripheral) GLP1 systems is not fully understood, making it difficult to pin down the discrete circuits involved in a systemic response to GLP1RAs. Recently, it was shown that peripheral GLP1 can suppress eating by activating nodose vagal afferents projecting to the nucleus tractus solitarius (NTS), independently from GLP1R neurones located in the brain.^47^ Similarly, we expand this concept to include a novel gut–carotid body–NTS pathway in which peripheral GLP1 sensing modifies centrally mediated sympathetic responses in metabolic homeostasis.^35^ Alongside their classical role in oxygen sensing, CBs are involved in whole body energy homeostasis and glucose sensitivity.^10,11^ It was shown that in healthy volunteers, hyperglycemic clamp augments the hypoxic ventilatory response (HVR),^13^ while glucose ingestion acutely increases SNA and is attenuated by desensitization of CBs using hyperoxia.^48^ We hypothesized that if in normal physiological conditions, GLP1 is released in response to increased levels of blood glucose, it could function to counteract the glucose mediated CB activation. Our data support this mechanism as Exendin-4 administration to the CB prevented hyperglycemia-evoked sympathetic sensitization in situ (Figure 5). If not the glucose, peripheral chemoreflex suppression by GLP1 could also function to prevent insulin or leptin mediated long-term chemosensory sensitization.^16,49^ In either case, alterations to the proposed mechanism would lead to aberrant sympathetic activation as seen in the SH rat (Figure 6D).

Finally, the presence of GLP1R in human CBs (Figure 6) opens a possibility that the antihypertensive/cardioprotective effect of GLP1RAs in T2D patients^42^ may in part be attributed to the inhibition of chemosensory drive from the CBs. Sympathetic activity^3^ and peripheral chemosensitivity^17^ have been shown to be increased while GLP-1 signaling is altered in T2D.^50^ Thus, the GLP1-CBs pathway may serve as a novel therapeutic target for controlling sympathetic activity in metabolic disease states as well as hypertension. Noteworthy, etiology of human hypertension is more diverse than that in spontaneously hypertensive rat in which it is primarily neurogenic. Further exploration of the discovered pathway in other model systems of hypertensive chemosensory sensitization (salt-sensitive, Goldblatt, diet-induced) is thus warranted. Taken together, this supports the undertaking of a proof-of-concept clinical study to test the inhibitory effects of GLP1-RAs on peripheral chemosensitivity.

A limitation of the current study is that we describe an acute effect of GLP1R-mediated peripheral chemoreflex suppression leading to reduced sympathetic activation. Whether prolonged treatment with GLP1RAs elicits the same effect remains to be demonstrated experimentally. Another limitation is that we pharmacologically stimulated GLP1R, and whether circulating GLP1 would elicit the same effect remains to be clarified. However, such a mechanism is not relevant for the treatment with GLP1RAs, which is when the antihypertensive/cardioprotective effects (and peripheral chemoreflex suppression) become important. Thus, our data support the use of GLP1RAs as potential therapeutics for the treatment of excessive sympathetic activity in T2D with hypertension; this remains to be tested.

## Article Information

### Acknowledgments

We are grateful to University of Bristol Genomics Facility, Wolfson Bioimaging Facility and ASU for their support and assistance in this work. We also thank Dr Ben J. Jones (Imperial College London) for the kind gift of CHO-K1-SNAP_GLP1R cells.

### Sources of Funding

British Heart Foundation (FS/17/60/33474); Health Research Council of New Zealand (19/687); BBSRC (BB/R016879/1); The Leverhulme Trust (RPG-2017-287); D.J. Hodson was supported by MRC (MR/N00275X/1 and MR/S025618/1) Project and Diabetes UK (17/0005681) Project Grants. This project has received funding from the European Research Council (ERC) under the European Union’s Horizon 2020 research and innovation programme (Starting Grant 715884 to D.J. Hodson).

### Disclosures

J. Broichhagen and D.J. Hodson have a licensing deal with Celtarys Research for production and supply of LUXendins and other fluorophore-conjugated peptidic pharmacophores

### Supplemental Material

Detailed Materials and Methods

Figures S1–S11

Tables S1–S10

Data Sets 1 and 2

References 51–55

## Supplementary Material


