# Supplementary figures and images for: GLP1R Attenuates Sympathetic Response to High Glucose via Carotid Body Inhibition
Source: Circ Res. 2022 Feb 1;130(5):694–707. doi: 10.1161/CIRCRESAHA.121.319874 (PMC8893134; doi:10.1161/CIRCRESAHA.121.319874)

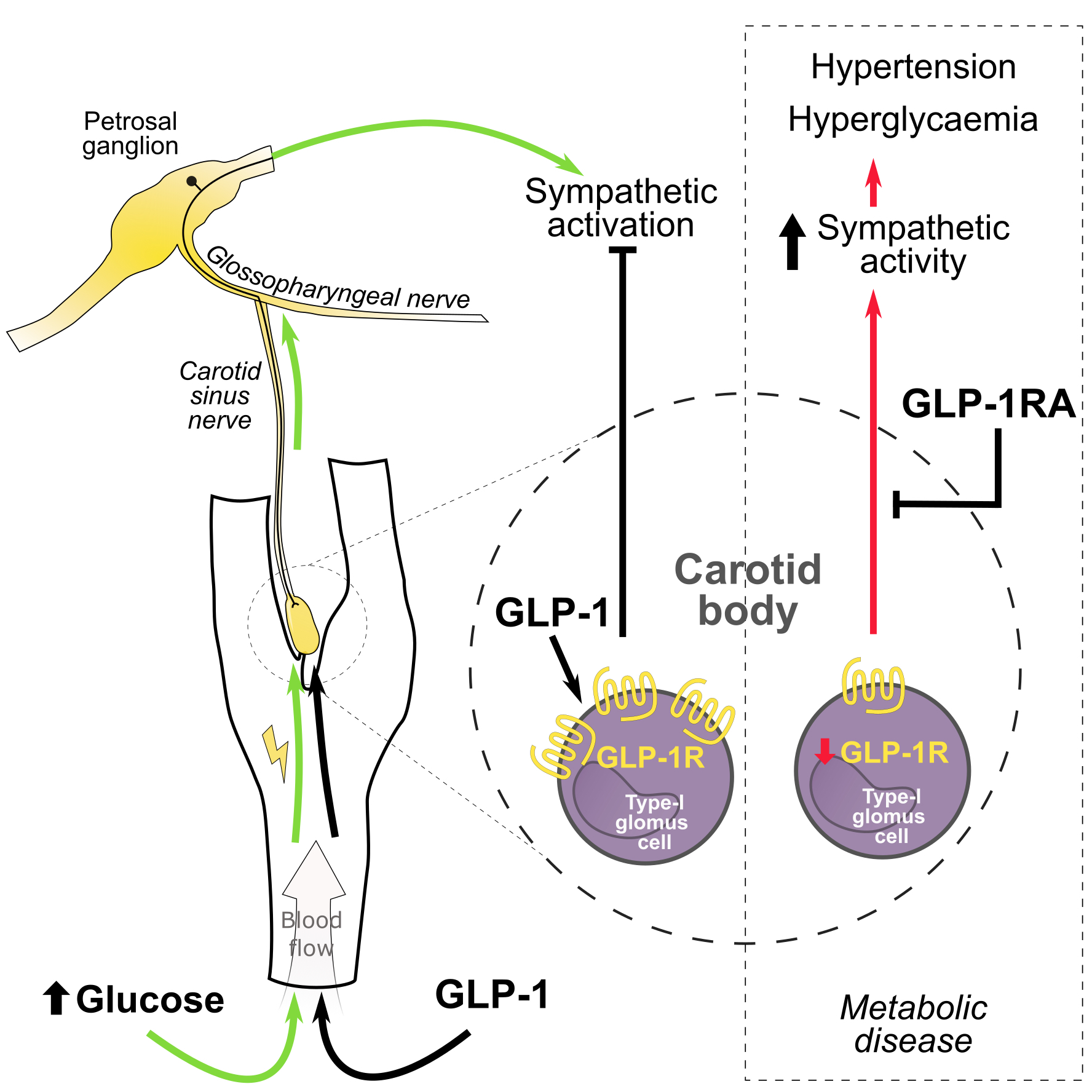

Supplement: Supplementary file 1 [file res-130-694-s001.jpg]
