# Supplementary material for: GLP1R Attenuates Sympathetic Response to High Glucose via Carotid Body Inhibition
Source: Circ Res. 2022 Feb 1;130(5):694–707. doi: 10.1161/CIRCRESAHA.121.319874 (PMC8893134; doi:10.1161/CIRCRESAHA.121.319874)
Supplement: Supplementary file 2 [file res-130-694-s002.pdf]

SUPPLEMENTAL MATERIAL

DETAILED MATERIALS & METHODS

Animal studies

Procedures involving use of laboratory animals were carried out independently in University of Bristol (United Kingdom), University of Belgrade (Serbia) and University of Auckland (New Zealand) with strict adherence to local rules and regulations. Aged-matched (12-14 weeks; or 4-6 weeks), male and female Spontaneously Hypertensive Rats (SHR), Wistar-Kyoto (WKY) rats or Wistar rats were used in the study. All procedures performed in University of Bristol were approved by the University of Bristol Animal Welfare and Ethical Review Board and performed under a Home Office UK license in accordance with the provision of the UK Animals (Scientific Procedures) Act (1986). All experiments performed in University of Belgrade were approved by the Faculty of Medicine, University of Belgrade, Experimental Animals' Ethics Committee. All procedures conformed to Directive 86/609/EEC and the University of Belgrade Guidelines on Animal Experimentation. All experiments performed in University of Auckland were approved and in accordance with the University of Auckland Animal Ethics Committee.

Animals were housed in conventional Tecniplast 1500 rat cages with controlled temperature (21 ± 2 °C), humidity (55 ± 10%) and 12 h light-dark cycle. Animals had unlimited access to food and water (*ad libidum*). Following transportation, animals were allowed to acclimatise to the housing facility for at least 7-days prior any experimental procedures. Due to hypothesis-free nature of the RNA-seq experiment, it was not possible to perform a priori power calculations. Group sizes for the RNA-seq experiment were based on prior work demonstrating that six biological replicates per experimental group are sufficient to identify >85% of differentially expressed gene.[51] Due to exploratory nature of the subsequent GLP1R characterization experiments, sample size and power calculation were based on: determining

response magnitude (based on previous reports or pilot experiments), data variance (our previous experience, published papers and pilot experiments), taking into consideration the number of drugs to be tested and drug dosages, and the expected technical success rate of each type of experiment. The minimum number of animals required per group were established based on data variance and anticipated goal (exploratory or confirmatory) of the experiment and ranged from n=5 to n=2 (**Table S6**). Due to highly technical nature of the studies and pronounced differences in basal physiological parameters between the strains, it was not possible to blind them. Animals were assigned to experimental groups based on strain, age and sex. Within study groups, animals are randomly allocated to control and experimental groups; through the University of Bristol Animal Management Information System (AMIS), animals are randomly assigned to cages by Animal Services Unit staff. The number of cages, and the number of animals per cage, is pre-set by the investigator on AMIS, but the allocation is independently performed. For experiments with only two groups (e.g. vehicle vs. drug treatment), randomisation is determined by the flip of a coin. We always randomise samples for post collection processing procedures. For example, when isolating samples, we always use the picking balls from bag principle to prevent processing bias. Only after the samples have been run do we reassign these samples to their treatment groups. To reduce technical error, the order of sample derivation and processing is random. Sampling times and protocols are standardised. Full details about the animals are summarised in **Table S6**.

Carotid body transcriptomic analysis and target gene characterisation

Sample collection

Animals were euthanised using pentobarbital sodium (300 mg kg^{-1}; Euthatal). Animals were intracardially perfused with ice-cold PBS and carotid bodies carefully excised from common

carotid artery bifurcation under a dissection microscope (**Figure S1a**). Carotid bodies were immediately placed into homogenizer tubes (Cat. # 9790B, Takara) and snap-frozen in liquid nitrogen. Samples were stored at -80°C until processed.

RNA-sequencing and data analysis

Samples were homogenized in QIAzol Lysis Reagent (Cat. # 79306, Qiagen). RNA was isolated using phenol-chloroform phase separation method from the aqueous phase using Direct-zol Microprep kit (Cat. # R2062, Zymo). On average, 80-150 ng of total RNA was recovered from a single carotid body. RNA integrity was determined using Agilent 4200-TapeStation System (Cat. # G2991AA; Agilent) using High-Sensitivity RNA ScreenTape (Cat. # 5067-5579; Agilent). Samples with RIN >7 and yield of >85 ng were selected for library preparation. RNA yields and integrity scores are summarised in **Table S7**. Sequencing libraries were constructed using TruSeq® Stranded mRNA library preparation kit (Cat. # 20020594, Illumina). Pooled libraries were sequenced on Illumina NextSeq500 platform using NextSeq 500/550 High Output v2.5 (150 Cycles) Kit (Cat. # 20024907; Illumina) on a paired-end multiplex mode at the University of Bristol Genomics Facility. Raw sequencing files were analysed using a bespoke data processing pipeline (**Figure S1b**). All samples passed initial quality control (FastQC) and no further filtering of the data was applied. RNA-STAR aligner was used to map reads to the rat reference genome (Ensembl Rnor_6.0, INSDC Assembly GCA_000001895.4, Jul 2014; Annotation – Rn6 Ensembl release Rnor_6.0.95) using paired-end mode and keeping other settings to default. On average, over 80% of total reads were uniquely mapped to the reference genome for each sample. Reads were counted at the gene level in a paired-end mode using featureCounts. This resulted in ~11 million uniquely-mapped, high-quality reads counted per sample on average. Initial principal component analysis (PCA) revealed an outlier, sample SHR4 right side, that

was removed from further data analysis (**Figure S1c**). Coincidentally, this sample had the lowest RIN score from all sequenced samples (**Table S7**). Differential expression analysis was performed using DESeq2 (v1.22.2) in R. Hypothesis testing was based on Wald-significance test with Benjamini-Hochberg multiple comparison correction. Relationship between statistical power to detect differential expression and generated read depth was modelled using SuperSeq (v0.0.0.99) package in R (**Figure S1d**).

ENSEMBL was used for genome annotations using biomaRt (v2.44.4), org.Rn.eg.db (v3.11.4), AnnotationDbi (v1.50.3) packages in R. Enrichment analysis was performed using ClusterProfiler (v3.10.1) and ReactomePA (v1.32.0) packages in R. Benjamini-Hochberg correction (pAdj < 0.05) was used for multiple comparison correction in enrichment analysis. Gene expression data were visualised using R.

Target gene validation using RT-qPCR

RT-qPCR validation was performed on pooled bilateral RNA samples for each individual animal. RNA was reverse transcribed using QuantiTec reverse transcription kit (#205311, Qiagen) using the included Qiagen RT Primer Mix containing optimised mix of RNA-specific oligo-dT and random primers. RT-qPCR was carried out in triplicates using PowerUp SYBR Green master mix (Cat. # A25742; Applied Biosystems) on a StepOnePlus Real-Time PCR system (Applied Biosystems, USA). RT-qPCR was carried using the default cycling settings consisting of 1) an initial denaturation at 95.0°C for 10min; 2) followed by an amplification step consisting of denaturation at 95.0°C for 15 sec and amplification at 60.0°C for 60 sec for x40 cycles; 3) at the end a melt-curve procedure was performed consisting of initial denaturation at 95.0°C for 15 sec, amplification at 60.0°C for 60 sec, and slowly rising the temperature by 0.3°C up to 95.0°C to generate the fluorescence melt curves of the resulting amplicons in each well, followed by a final denaturation step at 95.0°C for 15 sec.

No template controls (NTC) and melt curve analysis were included for each target on every plate. Eukaryotic translation initiation factor 4B (*Eif4b*; ENSRNOG00000010103) was used as a housekeeping control as this gene was found to be highly expressed and had lowest variance across all samples in the RNA-seq dataset. Expression fold-change is presented as $\log_2$ of $2^{-\Delta\Delta CT}$. Housekeeping genes were always run on the same plate as target genes. Hypothesis testing of the results was performed using ΔCT values. Full list of primers used in the study is provided in **Table S8**.

Immunofluorescence

Animals were perfused with ice-cold PBS and 4% (w/v in PBS) PFA solution (Cat. # P6148; Sigma). Common carotid artery bifurcations were dissected and placed in 4% PFA for additional 4-hour fixation at 4ºC. Samples were cryoprotected by an overnight immersion in 30% (w/v in PBS) sucrose (Cat. # S9378; Sigma) at 4°C. Samples were embedded in OCT-compound (Cat. # 14020108926; Biosytems) and serially sectioned using a cryostat to obtain 18μm sections in transverse orientation. Sections were mounted onto SuperFrost Plus microscopy slides (Cat. # 10149870; Thermo-Scientific), air-dried and stored at −80°C until used.

Briefly, sections were incubated in antigen retrieval solution (10mM Sodium Citrate, 1mM EDTA, 0.05% Tween-20, pH 6.0) for 20 min at 90ºC. Subsequently, sections were rinsed and incubated in 5% normal donkey serum (v/v), 5% normal goat serum (v/v) and 0.4% Triton X-100 (v/v) solution in PBS for 2 hours to block non-specific binding and tissue permeabilization. Next, sections were rinsed and incubated in primary antisera solution in 5% normal serum at 4ºC overnight. Primary antibodies: anti-GLP1R (1:500; ab218532; Abcam); anti-TH (1:500; MAB318; Chemicon); anti-GLP-1(7-36) amide (1:200; NBP2-23558, Novus Biologicals). Next, sections were rinsed and incubated in secondary antisera solution for 4

hours at r.t. Secondary antibodies: donkey anti-mouse AF488 (1:500; A21202; Invitrogen); goat anti-mouse AF567 (1:500; A11031; Invitrogen); donkey anti-rabbit AF568 (1:500; A100421; Invitrogen); and donkey anti-rabbit CF633 (1:500; SAB4600132; Sigma). Next, sections were incubated in *Lycopersicon esculentum* (tomato) lectin (1:100; DL-1174-1; Vector Laboratories) for 15 min at r.t. Lastly, sections were rinsed, incubated in DAPI solution (1:1000; Cat. # D9542; Sigma) for 10 minutes at r.t., mounted using Fluoroshield (Cat. # F6182; Sigma) and kept dark at 4ºC until imaged. Samples were imaged using Leica SP5-II AOBS confocal laser scanning microscope at the University of Bristol Wolfson Bioimaging Facility. Secondary antibody only controls were performed as part of immunohistochemistry protocol optimisation to validate absence of non-specific binding using target tissue sections. Secondary antibody only controls yielded consistent (negative) result. Representative images shown in Figure 2 was selected based on morphological similarity (distribution of glomus cell clusters and the amount of visible blood vessels) of CB sections between WKY and SHR strains. Images obtained from different animals are shown in **Figure S4**. Image processing and analysis was performed using ImageJ/Fiji.

Validation of anti-GLP1R primary antibody

Antibodies targeting GPCRs are known to have poor selectivity resulting in unreliable signal. Thus, we have thoroughly tested the specificity of anti-GLP1R primary antibody (ab218532, Abcam) in cultured mammalian cells and known sites of GLP1R expression in rat **(Figure S3)**. Briefly, HEK293 cells transiently over-expressing rat GLP1R or SNAP-tagged human GLP1R were stained with anti-GLP1R antibody (ab218532) and custom fluorescent SNAP-tag substrate label (SBG-TMR) to validate cross-reactivity with rat and human GLP1R **(Figure S3a)**[52]. Next, HEK293 co-transfected with rat GLP1R and shRNA targeting rat *Glp1r* mRNA, yielded a single 53-kDa GLP1R band while the signal was substantially

reduced by *Glp1r* knock-down (**Figure S3b**). Next, PC12 cells stably overexpressing rat GLP1R yielded a highly specific immunofluorescence signal that was absent in the control cells (**Figure S3c**). Next, we showed localised GLP1R immunoreactivity in know regions of GLP1R expression (internal positive control) such as hypothalamic arcuate and paraventricular nuclei (**Figure S3d**) and pancreatic islets of Langerhans (**Figure S5b**). We further assessed the efficacy of anti-GLP1R antibody (ab218532, Abcam) to detect human GLP1R compared to another anti-GLP1R antibody designed against human epitope (MAB3F52, Iowa DHSB) in CHO-K1 cells stably expressing human SNAP-tagged GLP1R (PSNAPGLP1, Cisbio)(**Figure S3e**). Ab218532 (Abcam) antibody selectively marked stably overexpressed human GLP1R, however, compared to MAB3F52 (Iowa DHSB) was less sensitive at detecting the human epitope.

Lastly, we demonstrate targeted knock-down of endogenous GLP1R expression in the hypothalamic supraoptic nucleus (SON) using anti-GLP1R (ab218532) antibody (**Figure S3f**). Here, GLP1R was knocked-down using stereotaxic AAV-mediated shRNA transduction as described previously[53]. shRNA targeting GLP1R was delivered unilaterally while contralateral hemisphere received a non-targeting (scrambled) shRNA control. Together, our results unequivocally demonstrate anti-GLP1R antibody (ab218532; Abcam) to be highly specific against rat and human GLP1R.

Cells used for antibody validation were: Human Embryonic Kidney 293AAV Cell Line (Source: Cell Biolabs, AAV-100); Rat adrenal medulla pheochromocytoma PC12 cells (Source: Prof Shelley J Allen, University of Bristol); and Chinese hamster ovary K1 cells overexpressing human SNAP-tagged GLP1R (Source: Dr Ben Jones, Imperial College London).

HEK-293AAV cells were cultured in DMEM (Sigma; D6546) supplemented with 10% (v/v) fetal bovine serum (FBS, Gibco), 2 mM L-glutamine (Gibco) and 100 unit/ml of penicillin-

streptomycin (Gibco). CHO-K1 cells stably expressing the human SNAP_GLP1R (Cisbio, Cat. # PSNAPGLP1) were culture in DMEM (D6546, Sigma) supplemented with 10% FBS (Merck), 1% non-essential amino acids (Merck), 25 mM HEPES (Merck), 500 µg/mL Geneticin (Fisher Scientific), 1% Pen/Strep (Fisher Scientific), and 2% L-glutamine (Thermo Scientific). Lastly, PC12 cells were cultured in DMEM (Sigma; D6546) supplemented with 10% (v/v) horse serum (Sigma; H1138), 5% (v/v) fetal bovine serum (FBS, Gibco), 2 mM L-glutamine (Gibco) and 100 unit/ml of penicillin-streptomycin (Gibco) in collagen IV coated plates. Cells were incubated at 37°C in a humidified incubator with 5% (v/v) CO_2. No mycoplasma contamination was detected during routine testing carried out throughout the facility.

Rat GLP1R coding sequence (CDS) was reverse transcribed from Sprague-Dawley tissue samples expressing GLP1R. Sequence was subcloned into a pcDNA3.1(+) vector and validated using Eurofins Genomics Sanger sequencing service. Rat GLP1R CDS contained three nucleotide substitutions (264C>T, 1326T>C, 1365A>G) that did not cause any change to amino acid sequence (silent mutations). shRNA cassette was generated and provided by Dr M.P. Greenwood (University of Bristol). shRNA sequence was cloned into pGFP-A-shAAV shRNA Vector (Cat. # TR30034; OriGene) and driven by U6 promoter as described previously[53]. Sequences used for non-targeting shRNA (5'AATTCTCCGAACGTGTCACGT) and rat Glp1r-shRNA (5'GCACGCATGAAGTCATCTTTG). To investigate cross-reactivity with human GLP1R cells were transfected with SNAP tagged human GLP1R (SNAP_hGLP1R; Cisbio, Cat. # PSNAPGLP1). Multiple reactions were performed in separate wells in parallel and treated as individual replicates.

For chemical treatments, cells were seeded onto tissue culture plates to 60%–70% confluence. For immunofluorescence experiments cell were seeded on poly-L-lysine-coated

coverslips and 6-well plates for immunoblotting and gene expression experiments. Transfections were performed using Lipofectamine™ LTX with PLUS™ Reagent (Cat. # A12621; Thermo Fisher Scientific, Waltham, MA, USA) following manufacturer's protocol. For immunocytochemistry, cells were seeded on poly-L-lysine-coated coverslips and fixed in cold 4% PFA (wt/v) solution in PBS (pH7.4) for 10 minutes. Cells were then permeabilised in 0.1% Triton X-100 (wt/v) solution in PBS for 8 minutes at room temperature. Next, non-specific binding of antibodies was blocked by incubating cells in 5% normal donkey solution (v/v) for 1 hour at room temperature. Coverslips were rinsed and incubated in primary antisera (Abcam, ab218532, 1:500; Iowa DHSB, MAB3F52, 1:30) overnight at 4°C. Next, cells were incubated in a solution containing secondary antibodies (donkey anti-rabbit IgG AF568, 1:500, A100421, Invitrogen; or donkey anti-rabbit IgG AF488, 1:500, A21206, Invitrogen) for 1.5 hours at room temperature. Finally, cells were rinsed and mounted using Fluoroshield anti-fading mounting medium (F6182; Sigma-Aldrich) and imaged.

For western blot analysis, cells were lysed using lysis buffer (50mM Tris-HCL pH7.4; 150mM NaCl; 5mM EDTA; 1% (v/v) Triton X-100) supplemented with protease inhibitors (Cat. # P8340; Sigma-Aldrich, St. Louis, MO, USA). Cell lysates were collected into 1.5mL tubes and incubated for 20 min at 4°C to allow full dissociation of cell membranes. Homogenates were centrifuged at 10,000rcf for 10 minutes at 4°C. Supernatant were aspirated and stored at -80°C until used. Protein samples were prepared in 4X Laemmli buffer (200mM Tris-HCl pH6.8; 8% (wt/v) sodium dodecyl sulfate; 0.4% (wt/v) bromophenol blue; 40% (v/v) glycerol). No heating was applied to avoid oligomerization following heat-induced protein denaturation. Proteins were resolved using SDS-PAGE on a 12% gel. Pierce™ Prestained Protein MW Marker (Cat. # 26612, Thermo Scientific™, Waltham, MA, USA) was used to assess protein size. Separated proteins were transferred to a 0.45 μm pore size PVDF membrane (Cat. # IPVH00010; Millipore, Burlington, MA, USA) and incubated in

5% (wt/v) bovine serum albumin (BSA, Cat. # A7906, Sigma-Aldrich) in Tris-buffered saline (150 mM NaCl; 20 mM Tris-HCl, pH 7.6) with 0.1% Tween 20 (TBS-T) for 1 hour at room temperature to block non-specific binding of the antibodies. Next, membrane was incubated in primary antisera (rabbit anti-GLP1R, 1:1000, ab218532, Abcam; or mouse anti-Gapdh, 1:5000; 60004-1-Ig, Proteintech) solution in 1% BSA (wt/v) in TBS-T overnight at 4°C. Next, membranes were thoroughly rinsed in TBS-T and incubated with secondary antibodies conjugated with horseradish peroxidase (rabbit anti-mouse IgG, A9044, Sigma; goat anti-rabbit IgG, A0545, Sigma) in 1% BSA solution in TBS-T for 1 hour at room temperature. Membranes were rinsed in TBS-T and signal was detected using SuperSignal West Dura Extended Duration (Cat. # 34075; Thermo Scientific™, Waltham, MA, USA) chemiluminescence substrate on a G:Box gel doc system (Syngene, Cambridge, United Kingdom). If needed, immunoblots were stripped in Restore™ Western Blot Stripping Buffer (Cat. # 21059; Thermo Scientific™, Waltham, MA, USA) and reprobed to assess the multiple proteins in the same blot.

For stable expression of rat GLP1R, PC12 cells were transfected using LTX Plus reagent with rat *Glp1r* CDS (described above) and selected for stable expression of the construct using G418 disulfate salt (Cat. # A1720, Sigma-Aldrich). 48 hours after transfection, cells were treated with 250 μg ml^{-1} G418 disulfate salt daily for 14 days. Surviving cells were seeded into six-well dishes and propagated. Stable-overexpression of GLP1R was assessed using RT-qPCR and immunofluorescence as described above. Immunofluorescence staining of hypothalamic arcuate and paraventricular nuclei were performed as described for the carotid bodies.

LUXendin645

Accessibility of GLP1R in the carotid bodies was assessed using LUXendin-645, a far-red fluorescent GLP1R antagonistic peptide label[23]. Briefly, animals were injected (s.c.) 5nmol of LUXendin-645 between the shoulder blades and 2 hours after drug administration animals were euthanised and intracardially perfused with ice cold PBS and 4% (w/v in PBS) PFA solution. Common carotid artery bifurcation, pancreas and brains were excised and processed as described for immunofluorescence. 40µm-thick frozen sections were prepared and stained using anti-TH antibodies and TL/LEL lectin. Heating and antigen-retrieval steps were omitted. Imaging was performed as described above using 10 mW Red He/Ne (633 nm) lasers to excite the conjugated LUXendin645 fluorophore. Noise2Void denoising algorithm was applied on images presented in the paper using N2V Fiji plugin.

Arterial blood pressure recordings *in vivo*

Surgery and experimental protocol

All surgical procedures were carried out as described previously[54]. Briefly, fine-bore polyethylene catheters (Smiths Medical International, UK) prefilled with heparinized saline (50 IU mL^{-1}) were inserted into right femoral artery (OD-0.96 mm, ID-0.58 mm) and right jugular vein (OD-1.09 mm, ID-0.38 mm) for arterial BP recording and agent administration, respectively. Hemodynamic parameters were recorded not earlier than 2 days post-surgery once animals were fully recovered. If the detected pulse pressure signal was weak and not suitable for arterial BP recordings (due to cannula clogging or twisting), and did not recover following cannula flushing with saline, these animals were omitted from further analysis and assigned to be used for blood sampling experiment (see Detailed Material & Methods section Quantitative measurement of plasma insulin and glucose).

Experimental protocol was designed to assess the acute effects of GLP1R agonism on carotid body chemosensitivity in conscious instrumented rats. Sodium cyanide was used to stimulate

the chemoreflex as this treatment provides a highly targeted, quantifiable, repeatable and temporally controlled stimulus originating from the carotid bodies[5,7]. Experiments were performed in awake, freely moving animals to avoid peripheral chemoreflex inhibition and respiratory depression induced by anaesthetics. To modulate GLP1R activity, we used a highly-selective, short-acting GLP1R agonist - Exendin-4 (Ex-4; Cat. # 1933, Tocris). An uninterrupted arterial BP recording was obtained for 15 minutes (**Figure 10a**). Animals were then administered a saline (sham) i.v. injection and 15 min later a bolus injection of 0.04% (m/v) NaCN (100µL i.v.) was administered via the jugular catheter to register reference chemoreflex response (baseline 1). A continuous recording of at least 15 min post-bolus was made to record any hemodynamic effects. After a washout period, this procedure was repeated to obtain a second reference chemoreflex response (baseline 2). After washout, animals were administered Ex-4 (5µg kg^{-1}, i.v.) and chemoreflex responses to NaCN stimulation were measured in a time-course experiment at 15-, 30-, 60- and 120-min following drug administration. The efficacy of Ex-4 was assessed in a separate experiment in which intravenous administration produced a surge in plasma insulin and glucose (**Figure S9a-b**)[38].

Data acquisition and analysis

Arterial blood pressure was recorded using a pressure transducer (P75 Hugo Sachs Elektronik) feeding into an inhouse BP-Complete software. Mean (MAP), systolic (SBP) and diastolic (DBP) arterial BP, pulse pressure (PP) and heart rate (HR) were derived from the raw arterial pulse pressure signal (**Figure S10**). For resting hemodynamic responses, each datapoint represents an average value across 2-5 minutes interval devoid of recording artifacts.

For SBP and HR variability analysis, derived SBP and HR signals were re-sampled at 20 Hz and subjected to nine-point Hanning window filter and linear trend removal as described previously[54]. Spectral analysis was performed using a fast Fourier transformation on 30 overlapping 4096 timepoint series spanning 204.8 seconds-long registration period. The power spectrum of systolic BP ($mmHg^2$) and heart rate (bpm^2) for 30 fast Fourier transform segments was then calculated and spectra were analysed up to 3 Hz, in total spectral volume (TV): 0.019–3 Hz; very low frequency (VLF): 0.019–0.2 Hz; low frequency (LF): 0.2–0.8 Hz; and high frequency (HF): 0.8–3Hz range. For SBP and HR variability analysis, each datapoint represents power spectra of the derived SBP and HR signal just before the drug administration (pre-saline, pre-Exendin-4) or the NaCN chemoreflex stimulus (pre-baseline1, pre-baseline2, 15 min, 30 min, 60 min, 120min) as indicated in grey areas in **Figure S11b**. Data for all SBP and HR variability spectra are summarised in **Figure S11**. Evaluation of the spontaneous baroreceptor reflex was performed using the method of sequences as detailed previously[54]. For BRS analysis, each datapoint represents the same interval that was used to calculate SBP and HR variability.

For the arterial BP response to peripheral chemoreflex stimulation using NaCN, the duration of the response was measured manually assessing the return of MAP, SBP, DBP and HR back to pre-stimulus level. Area under curve was measured for the defined duration of the response. To account for any changes in baseline hemodynamic parameters induced by Exendin-4 (**Figure S10**), measured chemoreflex-evoked responses are expressed as a percentage change from the pre-stimulus resting value. Vasopressor response was measured as the maximum SBP response (% increase) compared to the average pre-bolus resting SBP value over 1 minute period prior the stimulus administration. Bradycardic response was measured as the minimum HR response (% decrease) compared to average pre-bolus resting HR value over 1 minute period prior the stimulus administration. Pulse pressure response was

measured as the maximum PP response (% increase) during the post-peak period of the vasopressor response (devoid of any bradycardic episodes) compared to the average pre-bolus resting PP value over 1 minute period prior the stimulus administration. Original (raw) values of the chemoreflex-evoked BP responses that were used to calculate percentage change from baseline are presented in **Figure S9c-d**.

Quantitative measurement of plasma insulin and glucose

Blood samples were collected to assess the efficacy of Ex-4 by measuring its incretin effect. These animals underwent the same surgical procedure of jugular vein and femoral artery cannulation as outlined above. If detected pulse pressure signal was not suitable for arterial BP recordings, animals were assigned to be used for blood sampling experiment. Before the experiment, animals were starved overnight with unlimited access to water. At the start of the experiment, 400 µL of blood was collected from the arterial femoral catheter into disodium-EDTA tube (1.5 mg/mL, pH 7.5; E4884, Sigma-Aldrich) for baseline measure. Animals were then administered Exendin-4 (5 µg/kg; i.v.) via the jugular vein catheter and additional four 400µL blood samples were collected in a time-course experiment at 15-, 30-, 60- and 120-min following drug administration. Retracted blood was immediately replaced with an equal volume of pre-warmed isotonic saline at each point of the collection. Blood samples were centrifuged at 1500rcf for 10 minutes using a benchtop centrifuge (Eppendorf miniSpin Plus equipped with F-45-12-11 rotor, Hamburg, Germany). Plasma supernatant was transferred to a clean 1.5mL tube and stored at -20°C until used.

Plasma insulin and glucose was measured using rat insulin ELISA kit (80-INSRT-E01, Alpco, Salem, NH, USA) and colorimetric glucose assay (ab102517, Abcam), respectively, following manufacturer's instructions. Absorbance was read at 450nm using Bio-Rad iMark™ microplate absorbance reader (Bio-Rad Laboratories, Inc., Hercules, CA, USA).

Total plasma protein concentration was measured by Bio-Rad Protein Assay (Cat. # 500-0006, Bio-Rad, Hercules, CA, USA) using Bovine Serum Albumin (Cat. # A7906, Sigma-Aldrich) as standard. Plasma insulin and glucose concentrations were normalised to total plasma protein concentration to account for serial sample collection in which retracted blood was replaced with isotonic saline affecting plasma volume. Final insulin and glucose concentrations are expressed as ng mL^{-1} and mg dL^{-1}, respectively.

Arterially perfused *in situ* rat preparation

Working heart-brainstem preparation (WH-BP)

In situ working heart-brainstem preparation (WHBP) was set up as described originally[55]. Briefly, rats were deeply anesthetized (5% Isoflurane) until surgical plane was confirmed. Following sub-diaphragmatic bisection, the upper body was plunged into ice-chilled Ringer's solution, decerebrated at the pre-collicular level, and skinned. Preparation contained no gastrointestinal tract, adrenal glands, kidneys and pancreas (**Figure 3a**). Descending aorta was cannulated with a double-lumen catheter and perfused retrogradely via one lumen of the cannula using a peristaltic pump (Watson Marlow 505S) driving Ringer's solution containing an oncotic agent (1.25%, polyethylene glycol), which was gassed with carbogen (95% O_2 ,5% CO_2 mixture), warmed to 31°C, filtered (15mm pore diameter) and recirculated. A pressure transducer (Gould Ltd, USA) was connected to the second port of the double lumen cannula to monitor perfusion pressure (PP) in the aorta. Heart rate (HR) was derived from the inter R-wave interval of the electrocardiogram (ECG) recorded from subdermal electrodes placed into the front paws. Simultaneous recordings of activity from the phrenic (PNA) and thoracic sympathetic (tSNA) nerves between T3 and T5 were obtained using bipolar glass suction electrodes. These signals and the ECG were amplified (10 kHz, AM-Systems), filtered (50–1500 kHz, AM-Systems), digitized (1401 micro, CED), and recorded using

Spike2 (CED, UK) acquisition software. Spike2 was also used to analyse these variables together with heart rate and perfusion pressure. For the carotid sinus nerve (CSN) recordings, CSN was identified as a branch of the glossopharyngeal nerve projecting towards the CB; the nerve was sectioned distally and recorded using bipolar glass suction electrodes. Signals were amplified (10,000 x, A-M Systems model 1700), bandwidth filtered (10Hz–1 kHz, A-M Systems), digitised (10 kHz, Micro1401-3, CED), and recorded using software Spike2 (CED). Chemoreflex-evoked responses were calculated as an average of activity over 15-20s and compared to baseline level over the same epoch and expressed as a percentage change; data were compared before/after drugs and between rat strains. As the stability and quality of WHBP preparations is reflected by the consistency of PNA signals. If the PNA signals of a particular animal were not obtainable, no further experiment was performed, and that animal was excluded from the study.

Drug delivery to the carotid body

Internal carotid artery (ICA) was cannulated, and cannula tip located rostral to the CCA bifurcation (**Figure 3b**). Via this cannula, Exendin-4 (Cat. # 1933, Tocris) or Exendin-3 (9-39) amide (Cat. No. 2081, Tocris) were administered at a volume of 30µL using a Hamilton syringe. After accessing the carotid body, drugs were washed off via the external carotid branch (ECA) branch preventing it from accessing the brain and activating brain GLP1R. An arterial micro-clamp was placed on the left CCA to restrict aortic injections of NaCN (100µl, 0.04% (m/v) bolus) to the right carotid body (CB) only (**Figure 3a**). Contralateral common carotid artery was occluded to ascertain signal specificity.

Experimental protocol

Peripheral chemoreflex was tested using a bolus injection of 0.04% (m/v) NaCN (20µL). After 15 min of stable recording, NaCN chemoreflex stimulus was administered into the ICA to ensure the catheter was placed correctly such that drugs administered accessed the carotid body (**Figure 3e**). To allow modulators to be administered to the carotid body via the ICA, all subsequent chemoreflex stimulation was performed via bolus injections of 0.04% (m/v) NaCN (100µl) via the descending aorta while recording responses in HR, tSNA, PNA, and PP.

Two NaCN chemoreflex stimuli were administered and averaged to derive a reference control response value. Following drug administration into ICA a series of NaCN chemoreflex stimuli were administered at 2, 10, 20, and 30 minutes (**Figure 3e**). At the end of experiments in each animal, the perfusate pump was stopped, and the noise was recorded for 10 min. During data analysis, the noise was subtracted from stable SNA measurement before the stimulus at each datapoint representing baseline SNA. Following NaCN stimulus, the percentage change from baseline SNA was then calculated and compared between vehicle-treated and Ex-4 or Ex-3-treated Wistar and SH rat groups. In the context of the present study, our definition of a change in carotid body (CB) sensitization is based on the magnitude of the sympathetic nerve activity response to a supra-threshold, sub-maximal dose of sodium cyanide before and after Ex-4 or Ex-3 administration. We used a NaCN dose that was not maximal but supra-threshold (**Figure S8**). As identical NaCN stimulus was used to stimulate the arterial chemoreflex throughout the experimental protocol (**Figure 3e**), the difference in chemoreflex-evoked sympathetic nerve activity before and after Ex-4 or Ex-3 is described as change in carotid body sensitivity as described previously[6].

For the NaCN dose-response experiments (**Figure S8**), both PNA, tSNA and the right side CSN were simultaneously recorded, while the contralateral common carotid artery was kept unclamped. The chemoreflex was evoked with incremental doses of 0.04% (m/v) NaCN

(12.5, 25, 50, 75, 100, 125, 150, 200 μL) via the descending aorta through a side-line cannula connected to our double-lumen catheter; we allowed, at least, 5 min to elapse between injections. Therefore, the right CB yielded the recorded CSN discharge whilst the left CB evoked the quantified chemoreflex motor responses.

To investigate chemoreflex sensitisation in hyperglycaemic conditions, glucose concentration was increased in circulating Ringer's solution from 10 to 30 mmol L^{-1} (**Figure 5a, d**). Chemoreflex was stimulated by NaCN administration in the aortic cannula at 2, 10, and 20 minutes. Parallel experiments were performed by administering Exendin-4 (1mM) bolus into the ICA at the same time while adding glucose load into the Ringer's solution (**Figure 5a,d**). The objective of these experiments was to check whether glucose load increases resting and chemoreflex-evoked tSNA response, and whether Exendin-4 has any influence on SNA triggered through supplementary glucose.

Human studies

Under the direction of Prof Dainius H. Pauza, human carotid bodies were extracted from *post-mortem* remains of six individuals (4F/2M; White-European, Age: mean 66.8, range 50-81; BMI: mean 25.3, range 22.9-28.3). No participants were recruited. All participants provided informed consent and voluntarily donated their *post-mortem* remains for medical studies at the Institute of Anatomy, Lithuanian University of Health Sciences, legislated under the Republic of Lithuania People Burial Act (2007, No. X-1404; Article 17). All individuals died of natural causes. All procedures involving human tissue were carried out in accordance with local rules and regulations protecting participant anonymity and keeping prior medical history confidential. For five individuals, carotid bodies were microdissected bilaterally from the common carotid artery bifurcations and placed into RNA*later*™

stabilization solution (Cat. # AM7021; Invitrogen). From one individual, excised carotid bodies were placed in cold 4% PFA solution for 4 hours at 4°C and processed for immunohistochemical analysis at the laboratories of the Institute of Anatomy.

RNA from human carotid bodies was isolated using the same method described for animal tissue. RNA integrity was assessed using 4200-TapeStation platform with RNA ScreenTape (Cat. # 5067-5576; Agilent). Median RIN was 5.6 (range: 1.9–7.6). Reverse transcription and RT-qPCR was carried out as described for animal tissue. Gene expression is expressed as C_t values generated after running all three genes on a single plate. Primers used is provided in **Table S8**.

For immunohistochemistry analysis, samples were processed as described for animal tissue. Primary antibodies: anti-GLP1R (1:500; ab218532; Abcam), anti-TH (1:500; ab113; Abcam) and anti-UCHL1 (1:1000, ab8189, Abcam). Secondary antibodies: anti-rabbit Cyanine3 (1:400; AP182C; Sigma), anti-sheep AF488 (1:300; A-11015; Invitrogen), anti-mouse Cyanine5 (1:400; AP192S; Abcam). Sections were imaged using Zeiss LSM 700 laser-scanning confocal microscope equipped with a dual T-PMT sensor (Carl Zeiss). Images were taken using 40×/1.4 Plan Apochromat and 60×/1.46 αPlan Apochromat oil immersion objectives in ZEN Black SP1 software (Carl Zeiss). Image processing and analysis was performed using ImageJ/Fiji software.

Statistical analysis

For every dataset, data distribution was assessed by identifying extreme outliers (outliers were defined as values above Q3 + 1.5xIQR or below Q1 - 1.5xIQR and extreme outliers as values above Q3 + 3xIQR or below Q1 - 3xIQR), testing normality assumption by Shapiro-

Wilk test and visually inspecting data distribution using frequency histograms, distribution density and Q-Q (quantile-quantile) plots. Homogeneity of variance was tested using Levene's test. As each comparison consisted of a relatively small sample size (n=5-10) and thus normality of data distribution could not be accurately assumed, non-parametric tests were used for hypothesis testing. For qRT-PCR data: pairwise Mann-Whitney *U*-test followed by Benjamin-Hochberg multiple comparison correction was used. For arterial pressure *in vivo* and WHBP *in situ* data: Friedman test was used for the repeated-measures (withing-group) comparisons and the Kruskal-Wallis test was used for between-group comparisons. In cases where the requirements for a non-parametric repeated-measures Friedman test were not met due to missing datapoints (originating during the data collection e.g., twisting of the cannula during the response to chemoreflex stimulus) the Kruskal-Wallis test was applied to avoid further loss of data. For pairwise comparisons *post hoc* Dunn's test with Bonferroni correction was used. Statistical analysis was carried using Sigmaplot software (Systat Software Inc, USA) and 'stats' (v4.1.0) and 'rstatix' (v0.7.0) packages in R. Data visualisation was caried out using 'ggplot2' (v3.3.5) package in R.

Data availability

Original sequencing files and summarised read counts of RNA-seq experiment have been deposited to Gene Expression Omnibus (GEO) and are available under accession number: GSE178504

Supplementary Figures

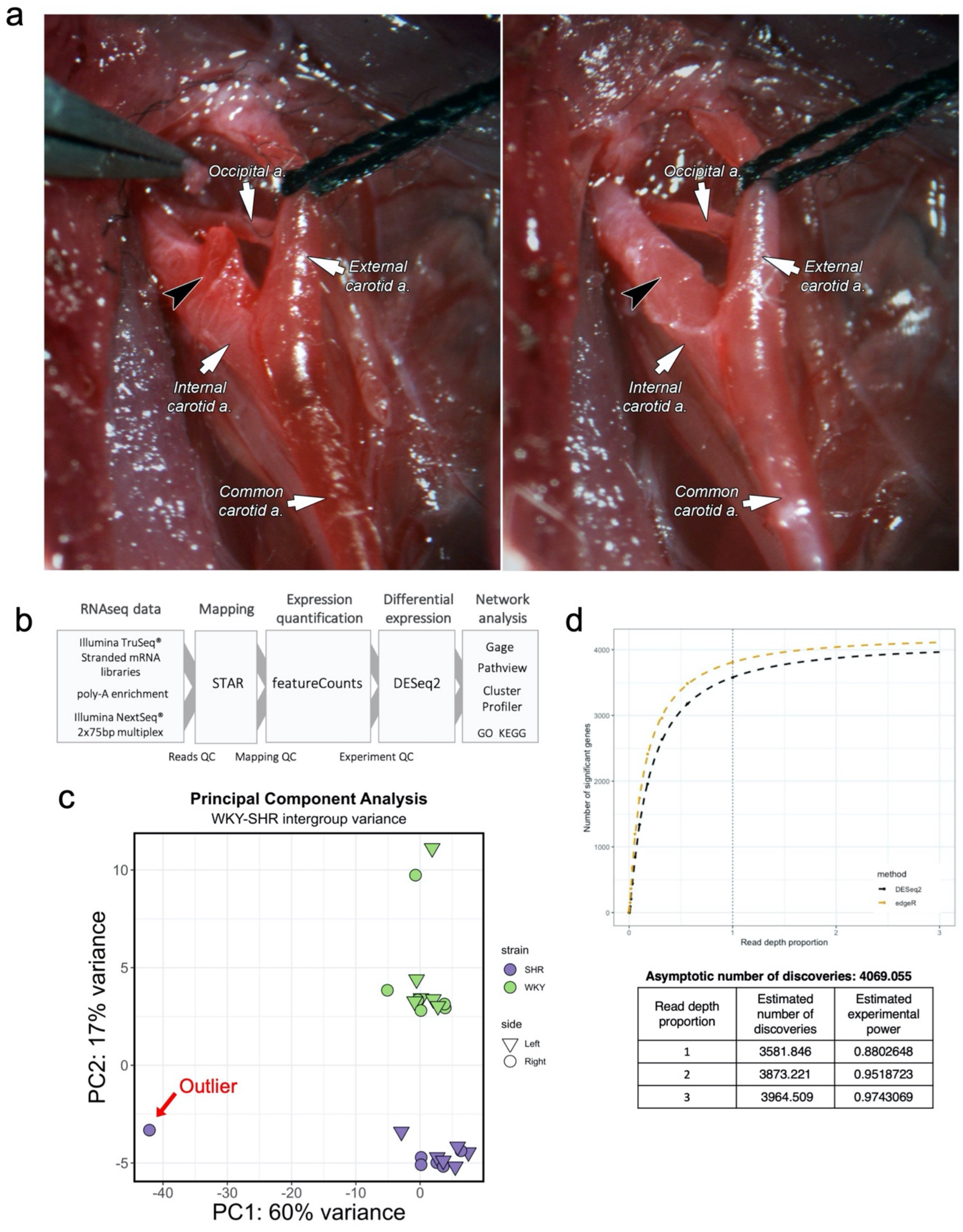

Read depth proportion	Estimated number of discoveries	Estimated experimental power
1	3581.846	0.8802648
2	3873.221	0.9518723
3	3964.509	0.9743069

Figure S1. Sample collection and transcriptomic analysis details

(**a**) Precise micro-dissection of rat carotid bodies from common carotid artery bifurcation area. Black arrowhead marks the carotid body. Note that samples used for gene and protein expression analyses were perfused and devoid of arterial blood.

(**b**) Data processing pipeline used for RNA-sequencing analysis.

(**c**) Initial principal component analysis (PCA) identified an outlier, sample SHR4 right side, that was removed from further data analysis. Coincidentally, this sample had the lowest RIN score from all sequenced samples (Table S7).

(**d**) SuperSeq model indicating read depth saturation for differential expression discovery using DEseq2 and edgeR methods. Read depth proportions indicate that the current experiment (indicated as 1 on the x-axis) achieved ~88.03% of estimated experimental power. DEseq2 method was selected for further analysis due to sample size suitability and popularity of this method permitting a direct comparison to other published datasets using the same method.

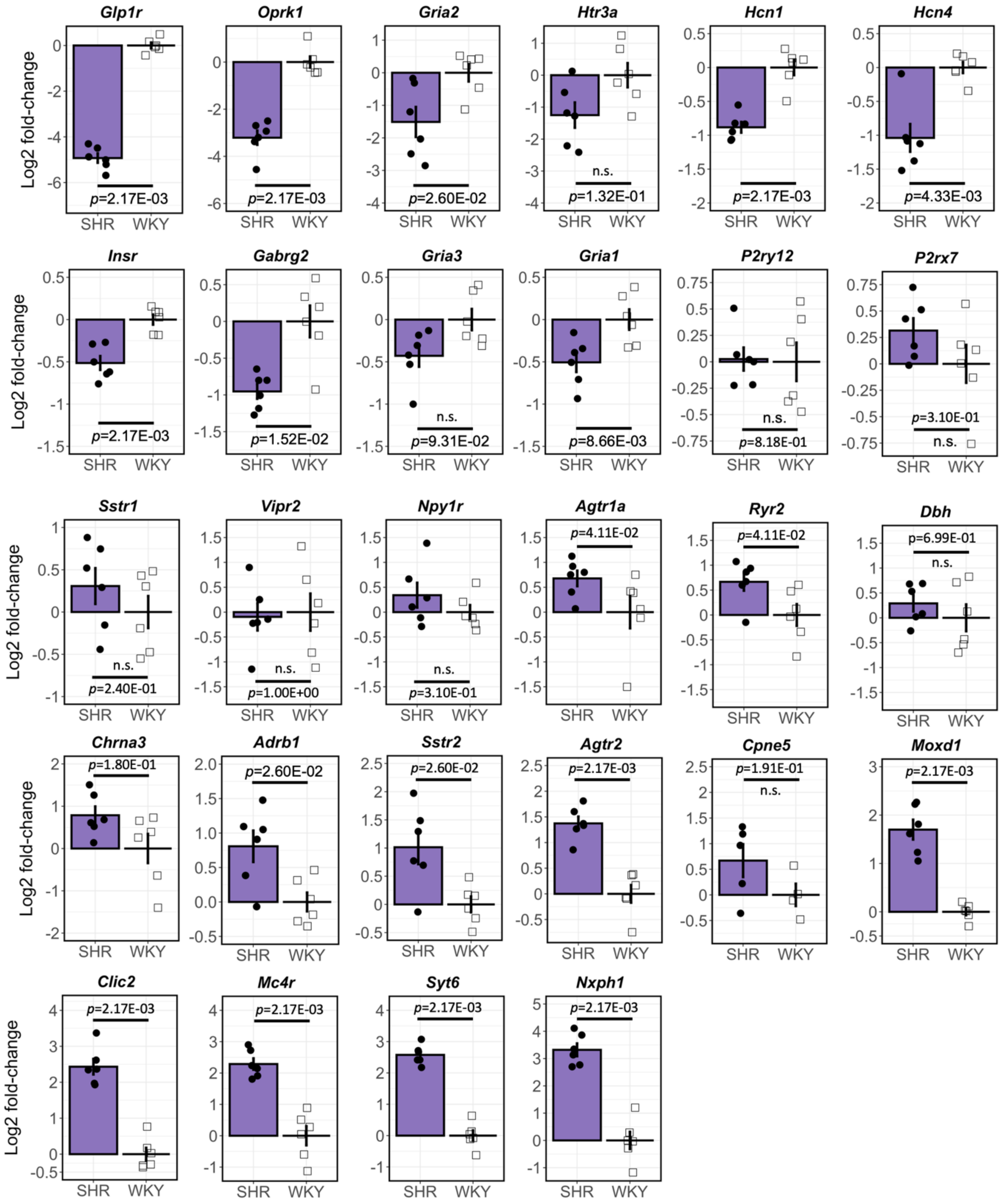

Figure S2. RT-qPCR validation of differentially expressed genes in carotid bodies of SH rats

Results of individual RT-qPCR reactions presented in **Figure 1f**. RT-qPCR validation was carried out in an independent cohort of age-matched (13-weeks old) male WKY/NHsd and SHR/NHsd rats. Expression fold-change is expressed as $\log_2$ of $2^{-\Delta\Delta CT}$ to permit direct

comparison to RNA-seq (DEseq2) results. Hypothesis testing was performed using ΔCt values. Means ± SEM. n=6. Mann-Whitney U-test.

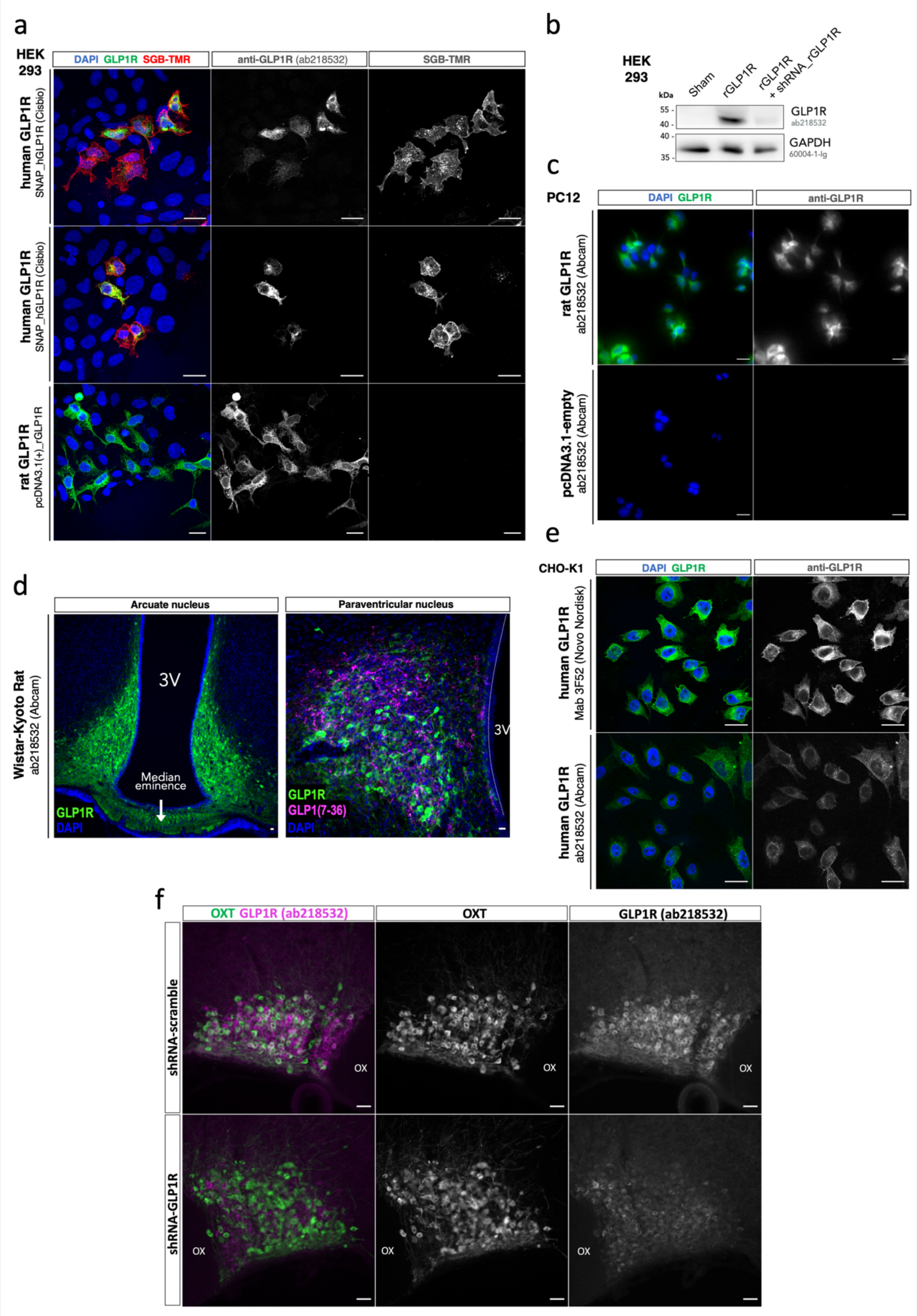

Figure S3. Specificity validation of anti-GLP1R primary antibody

Specificity of anti-GLP1R antibody (ab218532, Abcam) was tested in cultured cells and known sites of GLP1R expression.

(a) HEK293 transfected with rat GLP1R (pcDNA3.1(+)-rGLP1R-CDS) or SNAP tagged human GLP1R (SNAP_hGLP1R; Cisbio, Cat. # PSNAPGLP1). Cells were labelled with anti-GLP1R antibody (ab218532) and custom fluorescent SNAP-tag substrate label (SBG-TMR). Anti-GLP1R (ab218532) antibody selectively marked transiently over-expressed human GLP1R as indicated by colocalization with SNAP-tag substrate label SBG-TMR. Notably, anti-GLP1R (ab218532) was less sensitive at detecting human epitopes compared to rat GLP1R. n=1.

(b) HEK293 cells co-transfected with rat GLP1R and shRNA targeting rat GLP1R. Rat GLP1R overexpression yielded a single ~53-kDa band while the signal was substantially reduced in *Glp1r* knock-down (right lane). n=2. Data shown from one experiment.

(c) PC12 cells stably overexpressing rat GLP1R. Cells were transfected with a pcDNA3.1(+)-rGLP1R-CDS plasmid and selected for stable construct expression for 4-weeks using G418. PC12 cells stably producing rat GLP1R had a well-defined immunofluorescence signal compared to the negative control cells that underwent the same treatment. n=1.

(d) Immunofluorescence of GLP1R-positive neurones in rat hypothalamic arcuate and paraventricular nuclei representing internal positive control of the primary anti-GLP1R antibody (ab218532, Abcam). n=1.

(e) CHO-K1 cells stably expressing human SNAP_GLP1R (Cisbio). Ab218532 (Abcam) antibody selectively marked stably overexpressed human GLP1R. Notably, ab218532 (Abcam) antibody was less sensitive compared to MAB3F52 (Iowa DHSB) antibody specifically designed against the human epitope. n=1.

(f) Targeted knock-down of endogenous GLP1R expression in rat hypothalamic SON using AAV-mediated shRNA transduction. ShRNA-GLP1R was delivered unilaterally while

contralateral hemisphere received a non-targeting (scramble) shRNA control. ab218532 antibody specifically indicated reduced GLP1R production in the hemisphere receiving shRNA targeting *Glp1r*.

Representative images shown in plates **a, c, e** were selected to best represent the average signal across the slide. Scale Bar in all images – 20µm.

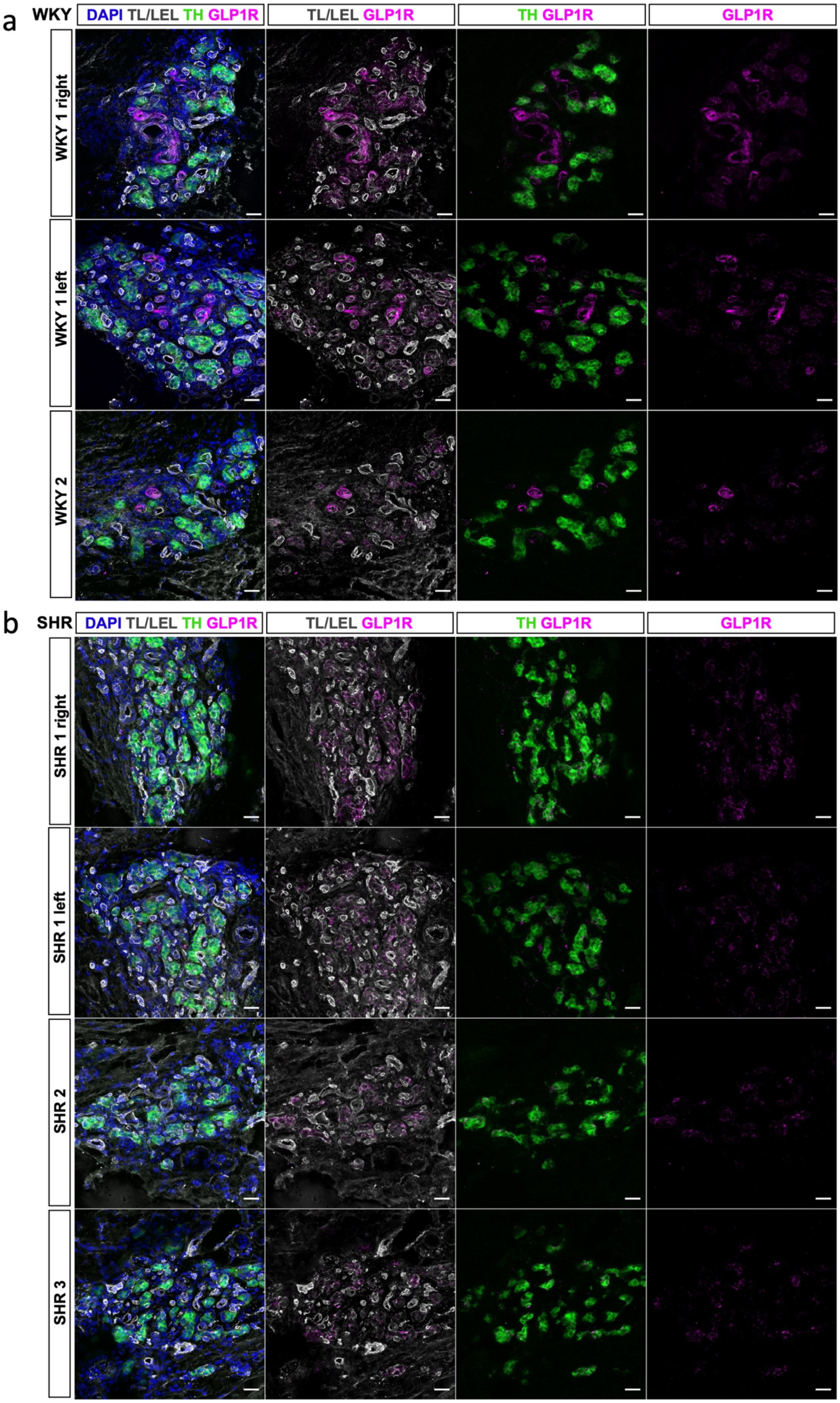
a
WKY
DAPI TL/LEL TH GLP1R
TL/LEL GLP1R
TH GLP1R
GLP1R
WKY 1 right
WKY 1 left
WKY 2
b
SHR
DAPI TL/LEL TH GLP1R
TL/LEL GLP1R
TH GLP1R
GLP1R
SHR 1 right
SHR 1 left
SHR 2
SHR 3

Figure S4. GLP1R localization in carotid bodies of SH and WKY rats

Immunofluorescence images showing GLP1R localization in carotid bodies of **(a)** WKY and **(b)** SH rats. GLP1R can be seen localised in Type-I glomus cell clusters (green) and in the media of blood vessels in the WKY rats. In the SH rats, GLP1R production is seen solely localised to the Type-I glomus cell clusters (green). Images shown in **(a)-(b)** were obtained from separate animals than that shown in Figure 2.

DAPI – 4′,6-diamidino-2-phenylindole (nuclear marker); TL/LEL – Lycopersicon esculentum (Tomato) Lectin (endothelial cell marker); TH – Tyrosine hydroxylase (chemosensory glomus cell marker); GLP1R – Glucagon like-peptide 1 receptor (. Images are representative of n=3. Scale bar – 20µm.

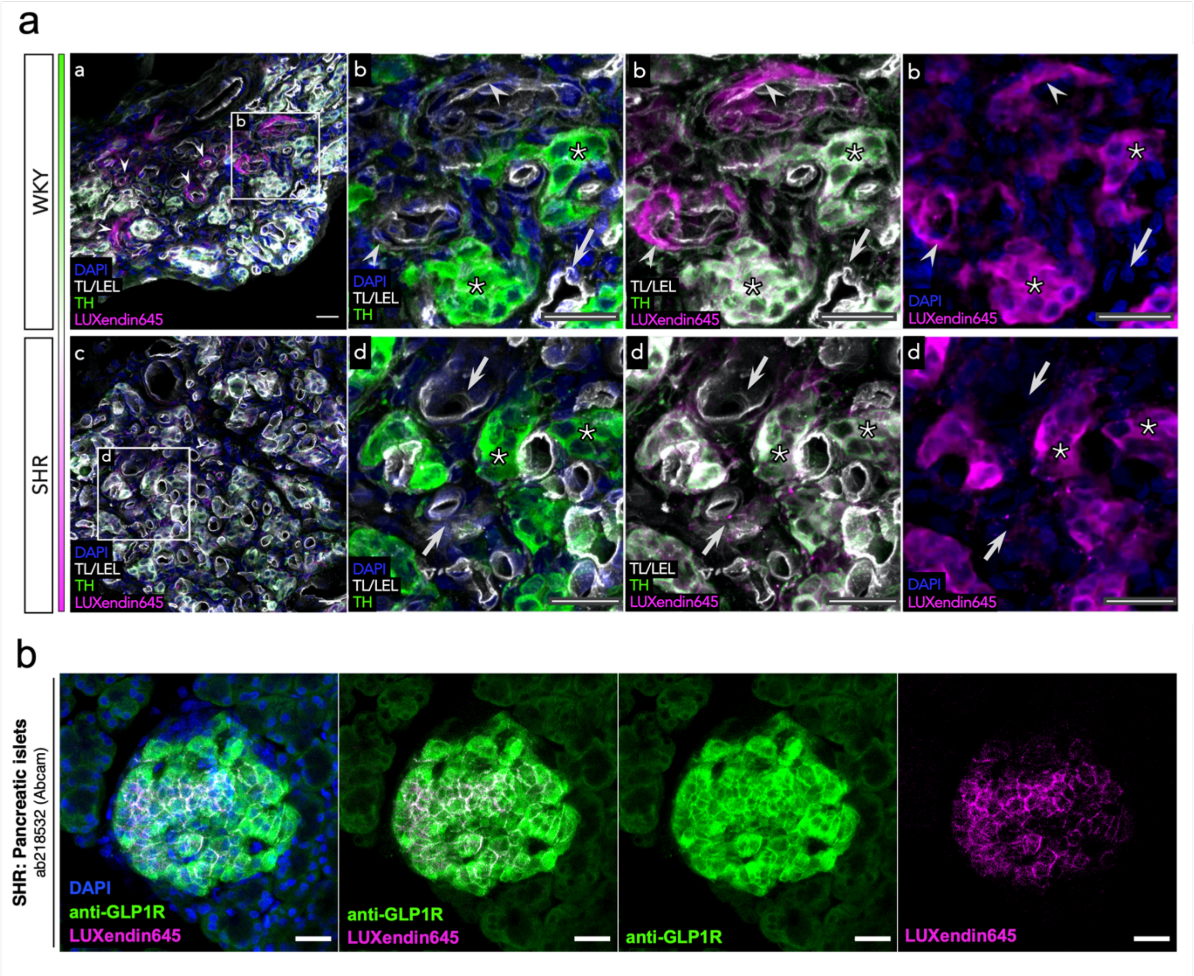

Figure S5. Accessibility of GLP1 receptors in carotid bodies of WKY and SH rats

(**a**) Transverse carotid body sections labelled with antagonistic peptide label LUXendin645 (5nmol) following subcutaneous injection. Dense capillary network is marked in grey, chemosensory glomus cell clusters are visualised in green. Intense labelling with LUXendin645 was localised to the chemosensory glomus cells in both WKY and SH rats. In contrast to normotensive WKY, no LUXendin645 labelling was associated with the TL/LEL-positive vascular component in the SH rat. Noise2Void algorithm was applied on LUXendin645 (magenta) signal to reduce background fluorescence. Representative images were selected to represent the average LUXendin645 labelling signal in the CBs across all WKY and SHR samples. The field of view was selected based on morphological similarity

(distribution of glomus cell clusters and the amount of visible blood vessels) of CB sections shown in **Figure 2**, permitting direct comparison between WKY and SHR strains.

(**b**) Pancreatic islet of an SH rat injected with LUXendin645 (5nmol; s.c.). Selective labelling of GLP1R-positive pancreatic β-cells colocalized with the antagonistic peptide label LUXendin645. Rabbit monoclonal anti-GLP1R (ab218532; 1:500) antibody was used to label GLP1R.

DAPI – 4′,6-diamidino-2-phenylindole (nuclear marker); TL/LEL – endothelial cell marker (tomato lectin). TH – tyrosine hydroxylase (1:500; MAB318). Asterisk – chemosensory glomus cell clusters; Arrowheads – blood vessels expressing GLP1R; Arrows – blood vessels devoid of GLP1R signal; Scale Bar 20µm. Images representative of n=2.

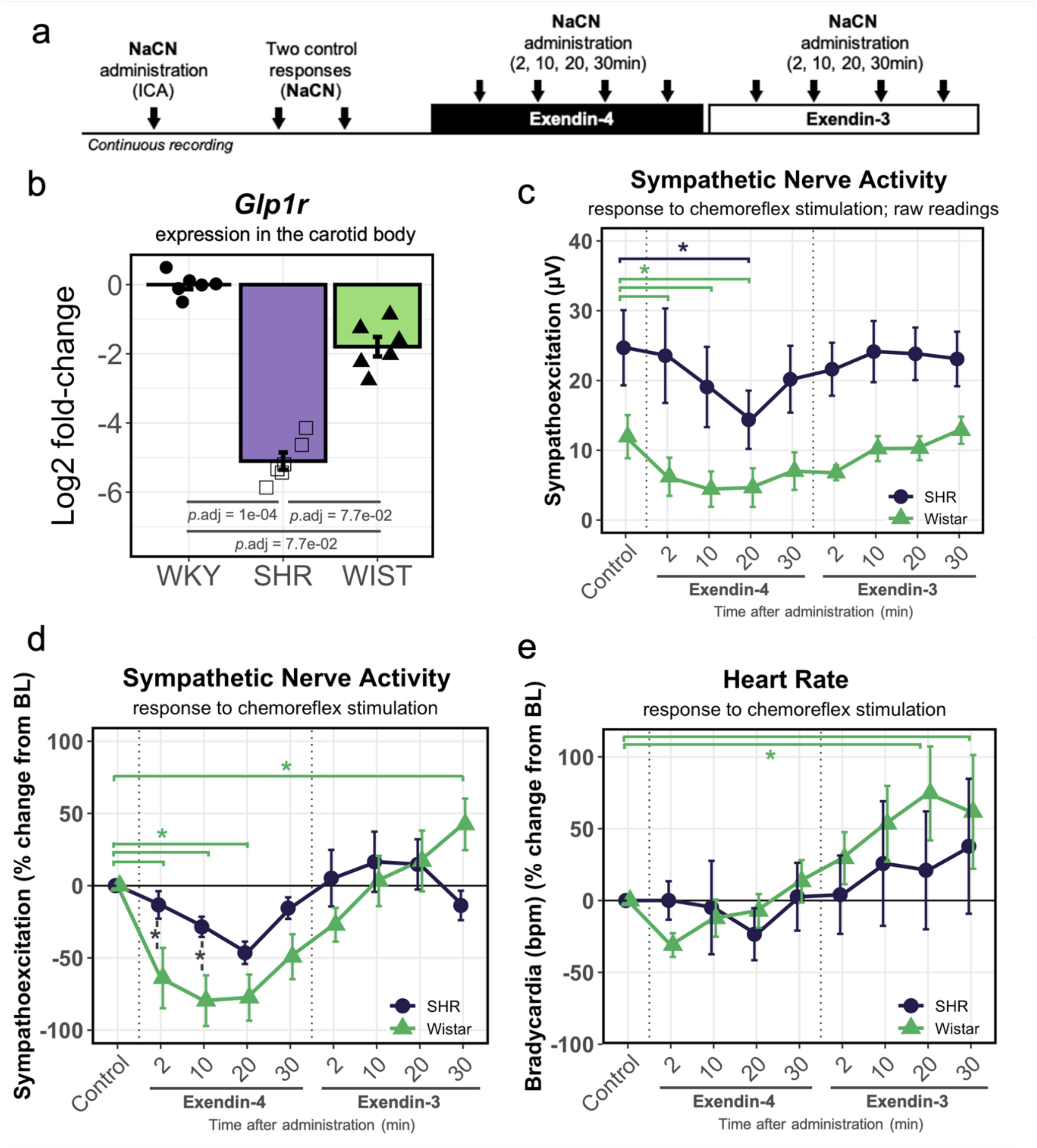

Figure S6. Time-wise response to GLP1R agonist (Exendin-4) and antagonist (Exendin-3) in Wistar and SH rats

(a) Protocol used for chemoreflex sensitivity assessment *in situ* when Exendin-4 and Exendin-3 were administered in sequence.

(b) *Glp1r* expression in carotid bodies of WKY, SH and Wistar rats. Values are presented as $\log_2$ of $2^{-\Delta\Delta CT}$ relative to WKY. n=6. Hypothesis testing was performed on ΔCt values using Kruskal-Wallis test, Dunn's post-hoc test (Bonferroni correction).

(c) Chemoreflex-evoked tSNA response shown in absolute (raw) values. Baseline values were significantly lower in Wistar relative to SH rats and remained lower throughout the protocol. Dotted lines indicate time of drug administration. n=8.

(d) Chemoreflex-evoked tSNA response expressed as percentage change compared to control response. In Wistar rats, Exendin-4 reduced chemoreflex-induced tSNA response while Exendin-3 counteracted this response to above baseline levels. Responses in SH rats were reduced. Dotted lines indicate time of drug administration. n=8.

(e) Chemoreflex-evoked bradycardic response expressed as percentage change compared to control HR response. No regular pattern of change in HR was seen in SH rats. In Wistar rats, Exendin-4 tended to decrease bradycardia and Exendin-3 significantly increased bradycardia 20 min after administration. n=8.

Non-parametric Friedman test (within-group) and the Kruskal-Wallis test (between-group), Dunn's post-hoc test (Bonferroni correction). Data shown as Mean ± SEM. *$p<0.05$

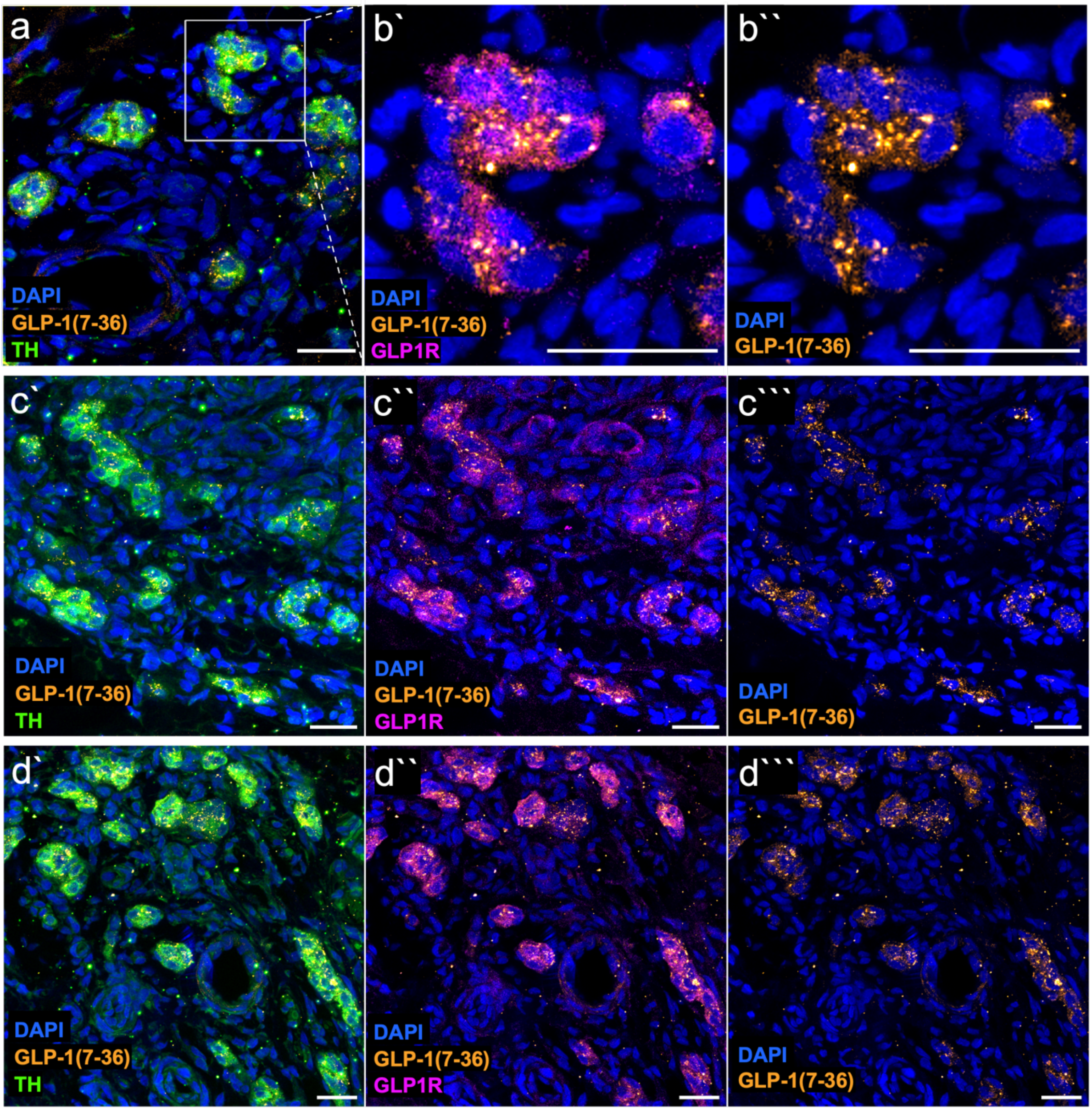

Figure S7. GLP1 peptide in Type-I glomus cell clusters

(a-d) GLP1 (7-36) amide (1:200; NBP2-23558) in carotid body sections of normotensive WKY rat. Structures resembling synaptic boutons were in close association with GLP1R expressing chemosensory, TH-immunoreactive glomus cell clusters.

DAPI – 4′,6-diamidino-2-phenylindole (nuclear marker); TH – tyrosine hydroxylase (1:500; MAB318); GLP1R – glucagon-like peptide 1 receptor (1:500; ab218532). Representative images were selected to represent the average GLP1 (7-36) amide immunostaining signal across all samples. n=2. Scale bar – 20µm.

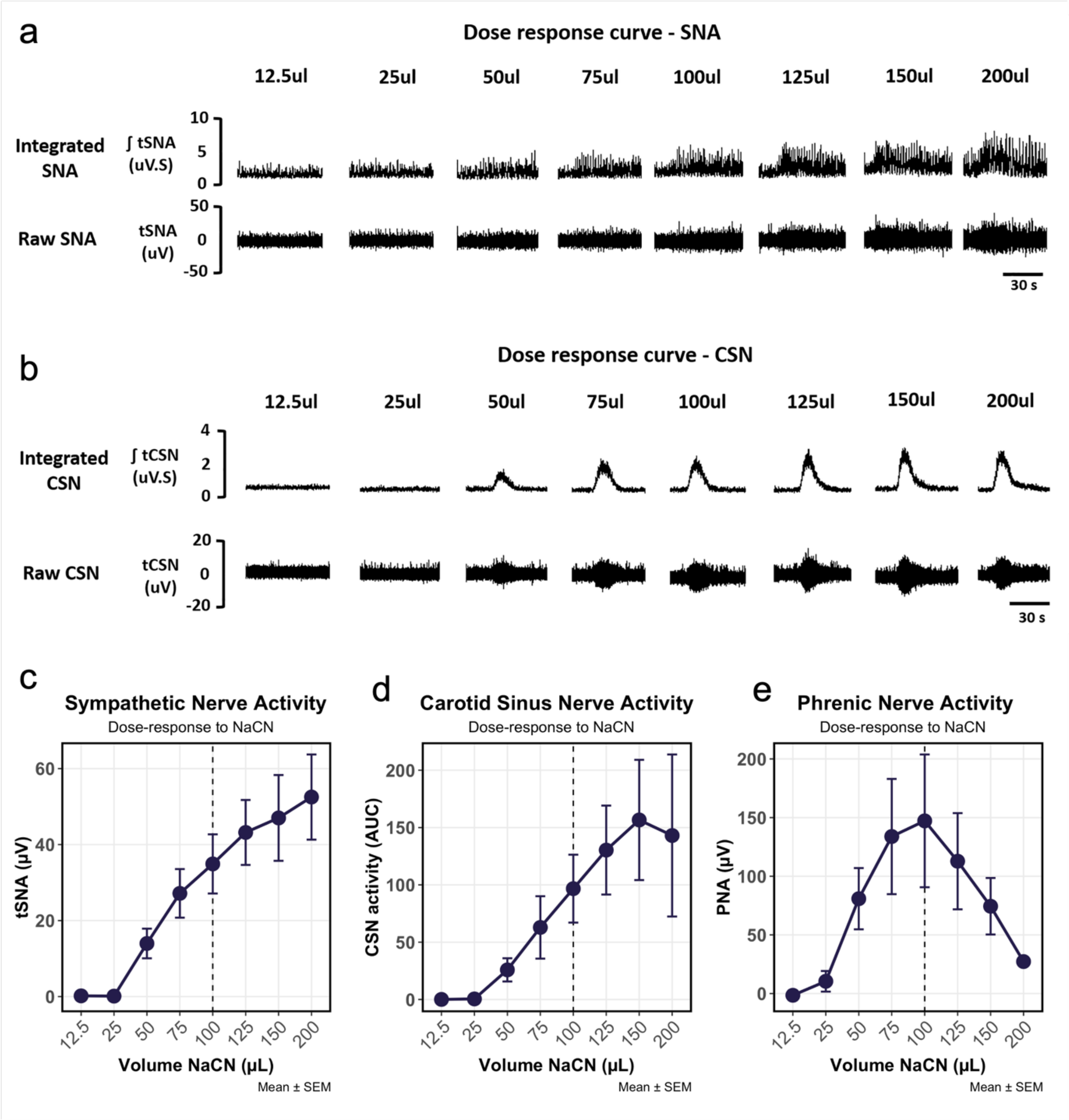

Figure S8. Sub-maximal, supra-threshold dose of NaCN used to assess chemoreflex sensitivity.

Representative **(a)** raw tSNA and integrated tSNA, and **(b)** raw CSN activity and integrated CSN activity response traces to increasing doses (bolus injection) of 0.04% (m/v) NaCN in Wistar rats. Traces of a single animal were selected to best represent the central tendencies (mean ± SEM value) obtained across all animals as shown in **c** and **d**. **(c)** tSNA (n=5), **(d)** CSN activity (n=4), **(e)** PNA (n=3) dose response curves to indicated doses (bolus injection) of 0.04% (m/v) NaCN. NaCN dose (100μL bolus; indicated by dashed line) selected for

WHBP experiments permitted the assessment of pharmacological effects on peripheral chemoreceptor sensitivity as this dose produces a supra-threshold, sub-maximal chemoreflex-evoked tSNA and CSN responses.

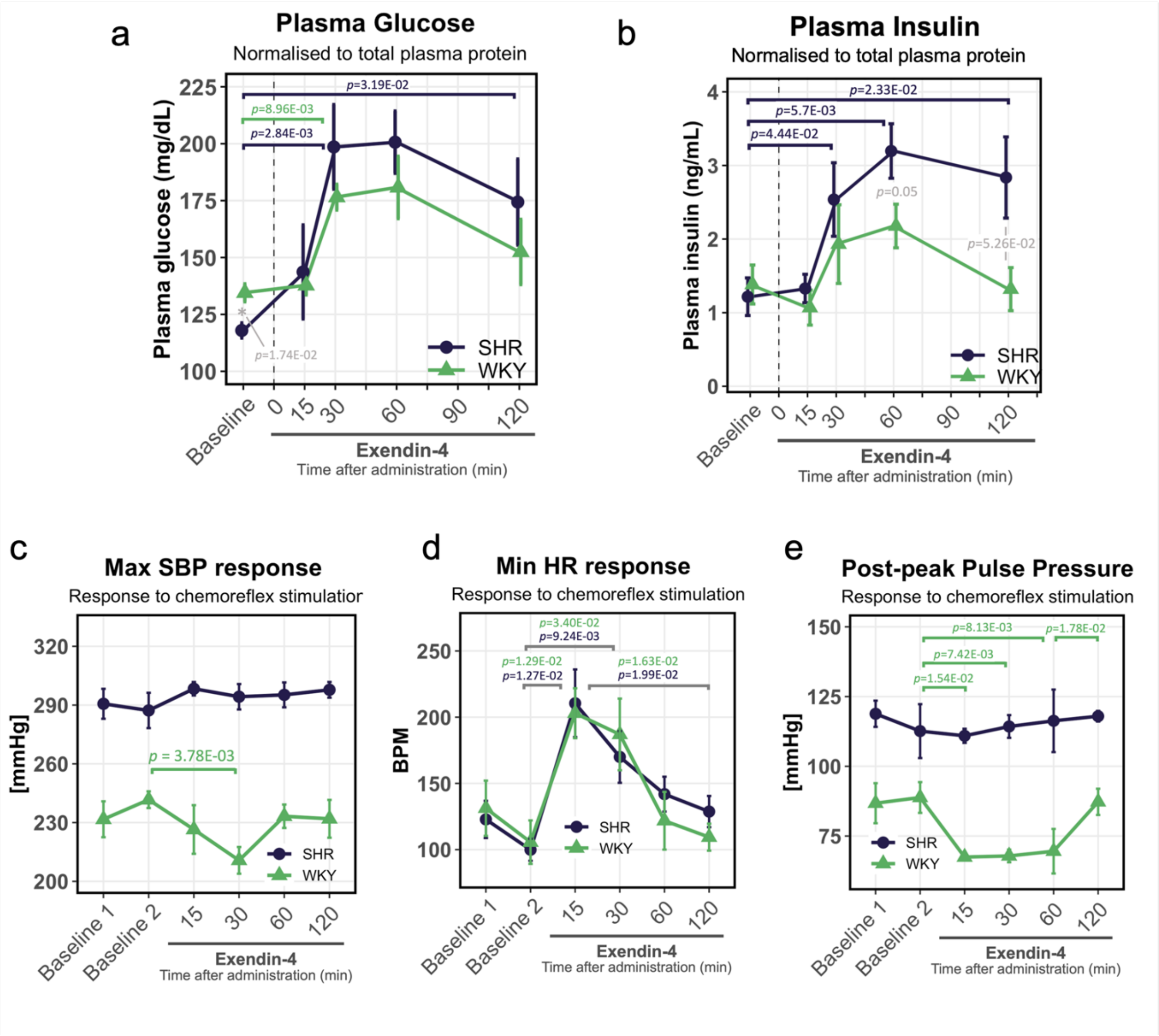

Figure S9. Direct measurements of Exendin-4 administration on chemoreflex-evoked arterial BP response in instrumented SH and WKY rats

(a) Plasma glucose and **(b)** insulin concentration in response to Exendin-4 (5 µg kg^{-1}, i.v) administration in overnight starved SH (n=4) and WKY (n=5) animals. Values shown are normalised to total plasma protein.

(c) Chemoreflex-evoked vasopressor response shown in absolute values (mmHg). Response was measure as the highest achieved systolic BP value following chemoreflex stimulation using NaCN. Values shown were used to calculate data presented in Figure 4i. n=6.

(d) Chemoreflex-evoked bradycardic response shown in absolute values (BPM). Response was measure as the lowest achieved HR value following chemoreflex stimulation using NaCN. Values shown were used to calculate data presented in Figure 4h. n=6.

(e) Chemoreflex-evoked pulse pressure increase response shown in absolute values (BPM). Response was measure as the highest achieved PP value during the post-peak period of the vasopressor response (devoid of bradycardic episodes) in response to NaCN stimulation. Values shown were used to calculate data presented in Figure 4j. n=6.

Kruskal-Wallis, Dunn's post-hoc test (Bonferroni correction). Data shown as Mean ± SEM. $^{*}p<0.05$, $^{**}p<0.01$

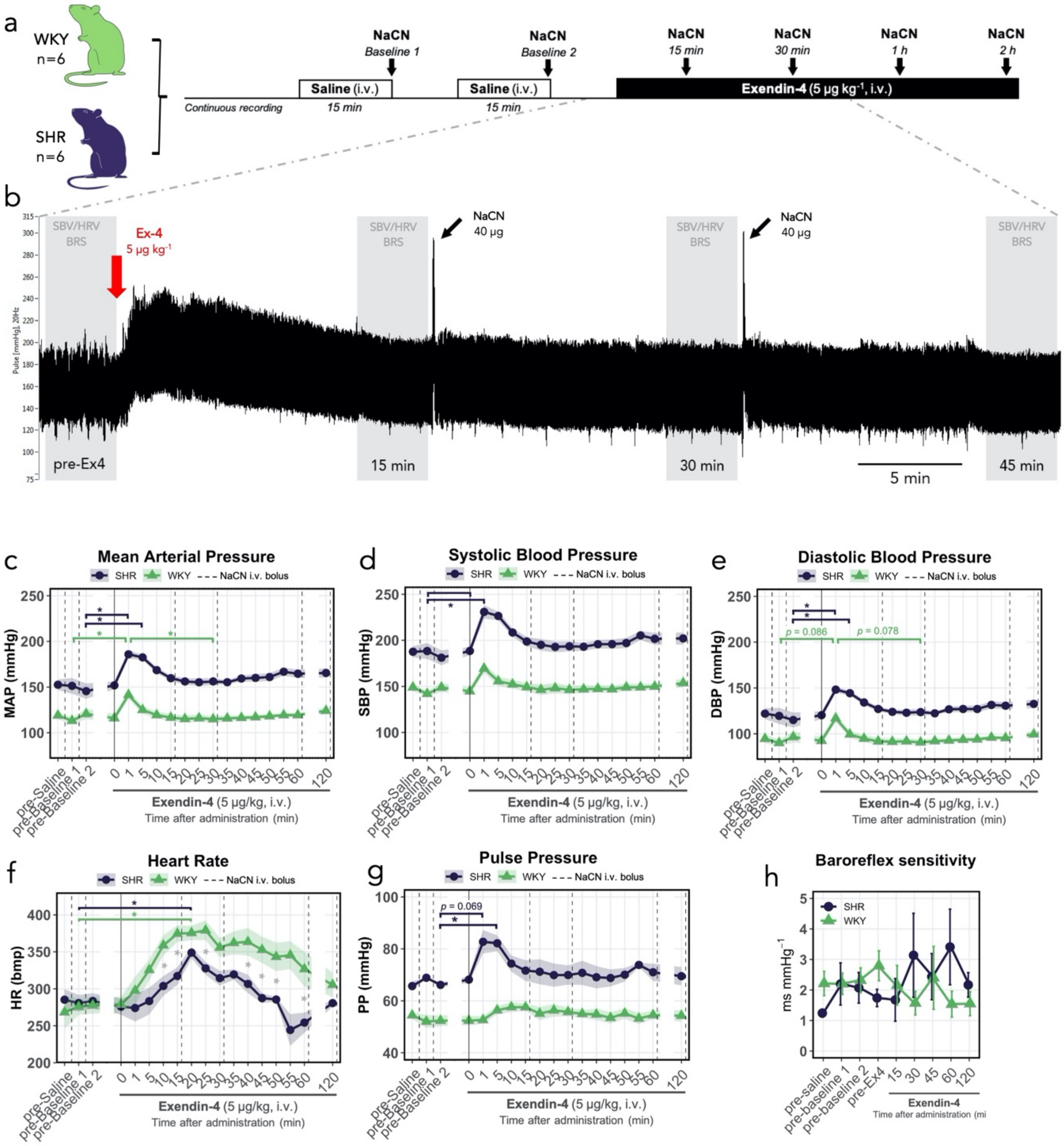

Figure S10. Resting haemodynamic responses to systemic Exendin-4 administration in instrumented SH and WKY rats

(a) Experimental protocol for testing peripheral chemosensitivity *in vivo*

(b) Representative pulse pressure trace following Exendin-4 administration (5 μg kg^{-1}, i.v) from a single SH animal. Red arrow indicates the time of Exendin-4 administration. Black arrows indicate chemoreflex stimulus (40μg NaCN bolus delivered in 100μL). Grey boxes

indicate intervals that were used to calculate systolic BP (SBV) and HR (HRV) variability and baroreflex sensitivity (BRS).

(c) Resting mean arterial pressure (MAP) response; **(d)** Resting systolic BP (SBP) response; **(e)** Resting diastolic BP (DBP) response; **(f)** Resting HR response; and **(g)** Resting pulse pressure (PP) response following Exendin-4 (5 µg kg^{-1}, i.v) administration. Dotted lines indicate times of chemoreflex stimulus (NaCN). Solid line marks Exendin-4 administration.

(h) Changes in baroreflex sensitivity following Exendin-4 (5 µg kg^{-1}, i.v) administration. Baroreceptor reflex sensitivity was assessed by the method of sequences.

n=6. Data shown as Mean ± SEM. Kruskal-Wallis, Dunn's post-hoc test (Bonferroni correction). *$p<0.05$

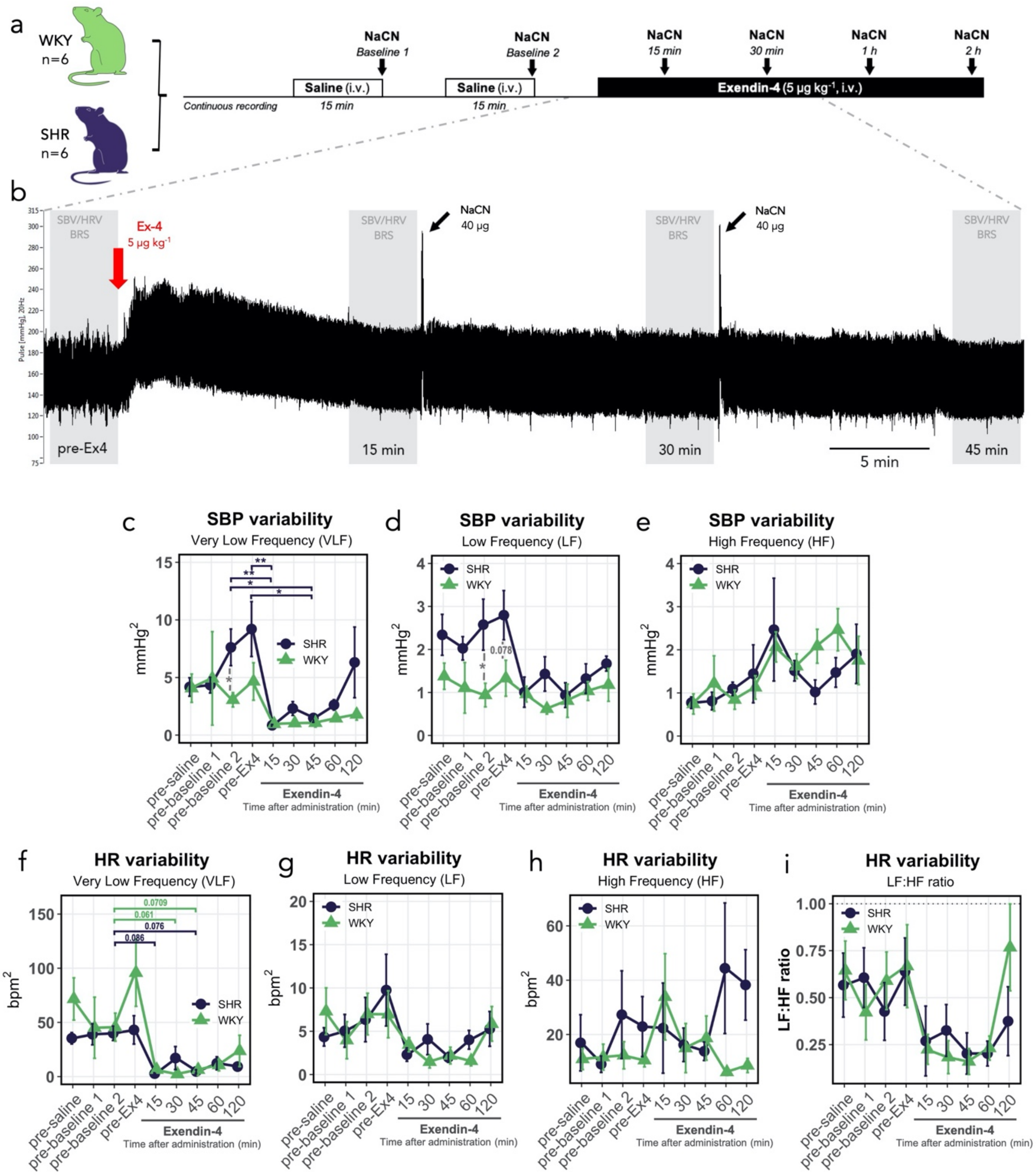

Figure S11. Systolic BP and HR variability in response to systemic Exendin-4 administration in instrumented SH and WKY rats

(a) Experimental protocol for testing peripheral chemosensitivity *in vivo*

(b) Representative pulse pressure trace following Exendin-4 administration (5 µg kg^{-1}, i.v) from a single SH animal. Red arrow indicates the time of Exendin-4 administration. Black arrows indicate chemoreflex stimulus (40µg NaCN bolus delivered in 100µL). Grey boxes

indicate intervals that were used to calculate systolic BP (SBV) and HR (HRV) variability and baroreflex sensitivity (BRS).

Systolic BP variability: **(c)** Very Low Frequency (VLF) domain, **(d)** Low Frequency (LF) domain, **(e)** High Frequency (HF) domain,

HR variability **(f)** Very Low Frequency (VLF) domain, **(g)** Low Frequency (LF) domain, **(h)** High Frequency (HF) domain, **(i)** Low Frequency – High Frequency ratio (LF:HF).

SBV and HRV were assessed for 3min 25sec intervals of derived systolic BP and HR signals just before stimulus administration as indicated by grey boxes in **(b)**. Data shown as Mean ± SEM. n=6. Kruskal-Wallis, Dunn's post-hoc test (Bonferroni correction). $*p<0.05$, $**p<0.01$

Supplementary Tables

Table S1. Effects of Exendin-4 on chemoreflex-evoked arterial blood pressure response

			Baseline		Exendin-4 (5 µg kg-1; i.v.)			
			1	*2*	*15 min*	*30 min*	*1 hour*	*2 hours*
**Response duration**	(s)	SHR	**20.07** ± 3.01	**18.79** ± 2.9	**6.11** ± 0.32	**7.93** ± 0.96	**12.96** ± 0.9	**16.37** ± 1.1
		WKY	**16.94** ± 4.02	**17.84** ± 3.35	**5.99** ± 0.65	**7.55** ± 1.53	**9.46** ± 1.54	**14.46** ± 2.14
	% change compare to baseline	SHR	–	–	-68.54	-59.18	-33.27	-15.76
		WKY	–	–	-65.55	-56.56	-45.59	-16.85
**Response AUC**	(AU)	SHR	**3592** ± 516.62	**3378.33** ± 424.84	**1202.5** ± 76.55	**1529.16** ± 170.97	**2574.16** ± 204.61	**3194.16** ± 228.91
		WKY	**2128.6** ± 628.12	**2440.33** ± 622.61	**867.2** ± 239.04	**1029.16** ± 282.01	**1328.16** ± 484	**2189.6** ± 620.81
	% change compare to baseline	SHR	–	–	-65.49	-56.12	-26.13	-8.34
		WKY	–	–	-62.03	-54.94	-41.86	-4.15
**Bradycardic response**	Decrease from pre-bolus HR (%)	SHR	**-62.66** 3.23	**-68.82** 2.66	**-42.67** 7.9	**-52.38** 5.55	**-54.62** 4.34	**-58.83** 4.69
		WKY	**-56.89** 6.6	**-66.01** 6.04	**-48.22** 5.5	**-52.81** 6.4	**-67.13** 5.43	**-69.69** 2.36
	% change compare to baseline	SHR	–	–	-35.09	-20.32	-16.91	-10.51
		WKY	–	–	-21.52	-14.06	9.24	13.4
**Vasopressor response**	Increase from pre-bolus SBP (%)	SHR	**55.35** ± 3.75	**54.48** ± 1.63	**50.49** ± 2.79	**52.22** ± 2.03	**47.15** ± 2.79	**47.9** ± 3.29
		WKY	**63.25** ± 6.44	**64.94** ± 5.36	**56.01** ± 6.37	**44.43** ± 5.1	**55.74** ± 3.73	**51.14** ± 4.35
	% change compare to baseline	SHR	–	–	-8.06	-4.91	-14.15	-12.78
		WKY	–	–	-12.62	-30.69	-13.04	-20.21
**Pulse Pressure increase**	Increase from pre-bolus PP (%)	SHR	**71.7** ± 6.8	**73** ± 10.89	**58.22** ± 10.8	**65.32** ± 9.51	**63.67** ± 12.88	**71.88** ± 9.39
		WKY	**67.4** ± 9.89	**68.92** ± 12.27	**20.9** ± 4.78	**22.54** ± 4.78	**27.42** ± 14.85	**62.15** ± 10.69
	% change compare to baseline	SHR	–	–	-19.53	-9.72	-12.00	-0.65
		WKY	–	–	-69.34	-66.93	-59.77	-8.82

Source data used in Figure 4. Key: AUC – area under curve.

Table S2. Effects of Exendin-4 on basal and chemoreflex-evoked tSNA

		**Exendin-4**					**Vehicle**				
		Control	*2 min*	*10 min*	*20 min*	*30 min*	*Control*	*2 min*	*10 min*	*20 min*	*30 min*
Chemoreflex evoked tSNA: Change from baseline (%)	Wistar	**0.00**	**-57.77**	**-79.60**	**-77.49**	**-49.20**	**0.00**	**-5.46**	**-4.93**	**-0.91**	**-6.02**
		–	± 23.04	± 17.54	± 15.95	± 15.57	–	± 6.05	± 12.05	± 15.53	± 18.33
	SHR	**0.00**	**-13.32**	**-28.50**	**-46.47**	**-15.52**	**0.00**	**2.82**	**6.70**	**16.67**	**14.02**
		–	± 9.54	± 7.01	± 7.74	± 7.48	–	± 3.24	± 5.33	± 7	± 15.33
Resting tSNA: Change from baseline (%)	Wistar	**0.00**	**-7.90**	**-10.70**	**-11.44**	**–**	**0.00**	**-1.86**	**1.86**	**10.49**	**–**
		–	± 4.36	± 5.22	± 6.13	–	–	± 0.46	± 5.11	± 4.91	–
	SHR	**0.00**	**-2.73**	**-1.62**	**2.04**	**–**	**0.00**	**0.85**	**4.12**	**8.03**	**–**
		–	± 1.03	± 1.72	± 4	–	–	± 0.09	± 3.89	± 4.18	–

Source data used in Figures 3c-i. Values presented represent mean ± SEM.

Table S3. Effects of Exendin-3 (9-39) on basal and chemoreflex-evoked tSNA

		**Exendin-3**					**Vehicle**				
		Control	*2 min*	*10 min*	*20 min*	*30 min*	*Control*	*2 min*	*10 min*	*20 min*	*30 min*
Chemoreflex evoked tSNA: Change from baseline (%)	Wistar	**0.00**	**26.23**	**23.49**	**33.82**	**28.28**	**0.00**	**-5.46**	**-4.93**	**-0.91**	**-6.02**
		–	± 15.16	± 18.18	± 26.46	± 31.58	–	± 6.05	± 12.05	± 15.53	± 18.33
	SHR	**0.00**	**45.94**	**49.63**	**46.95**	**40.79**	**0.00**	**2.82**	**6.70**	**16.67**	**14.02**
		–	± 17.4	± 22.34	± 22.91	± 26.16	–	± 3.24	± 5.33	± 7	± 15.33
Resting tSNA: Change from baseline (%)	Wistar	**0.00**	**6.57**	**19.93**	**23.43**	**–**	**0.00**	**-1.86**	**1.86**	**10.49**	**–**
		–	± 3.03	± 10.88	± 12.53	–	–	± 0.46	± 5.11	± 4.91	–
	SHR	**0.00**	**5.49**	**11.36**	**20.83**	**–**	**0.00**	**0.85**	**4.12**	**8.03**	**–**
		–	± 1.38	± 3.38	± 5.01	–	–	± 0.09	± 3.89	± 4.18	–

Source data used in Figures 3d-j. Values presented represent mean ± SEM.

Table S4. Time-wise raw tSNA, chemoreflex-evoked tSNA and chemoreflex-evoked bradycardic responses to Exendin-4 and Exendin-3 in Wistar and SH rats

			**Exendin-4**				**Exendin-3**			
		Control	2 min	10 min	20 min	30 min	2 min	10 min	20 min	30 min
Raw tSNA (μV)	Wistar	**11.95**	**6.20**	**4.43**	**4.65**	**7.00**	**6.78**	**10.23**	**10.29**	**12.87**
		± 3.09	± 2.73	± 2.53	± 2.76	± 2.69	± 1.08	± 1.79	± 1.71	± 1.94
	SHR	**24.69**	**23.55**	**19.07**	**14.37**	**20.18**	**21.61**	**24.14**	**23.82**	**23.08**
		± 5.38	± 6.76	± 5.75	± 4.17	± 4.79	± 3.79	± 4.38	± 3.76	± 3.91
Chemoreflex evoked tSNA: Change from baseline (%)	Wistar	**0.00**	**-63.95**	**-79.60**	**-77.49**	**-49.20**	**-27.09**	**3.26**	**17.11**	**42.45**
		–	± 20.89	± 17.54	± 15.95	± 15.57	± 11.65	± 17.42	± 21.05	± 17.86
	SHR	**0.00**	**-13.32**	**-28.50**	**-46.47**	**-15.52**	**5.21**	**16.54**	**14.73**	**-13.69**
		–	± 9.54	± 7.01	± 7.74	± 7.48	± 19.58	± 20.84	± 17.37	± 10.21
		**0.00**	**-31.01**	**-12.30**	**-7.18**	**13.39**	**26.22**	**45.68**	**65.76**	**57.84**

Chemoreflex - evoked bradycardia: Change from baseline (%)	Wistar	–	± 8.21	± 12.91	± 11.67	± 14.75	± 16.04	± 24.12	± 29.67	± 34.49
	SHR	**0.00**	**0.07**	**-4.86**	**-23.48**	**2.66**	**4.03**	**25.74**	**21.03**	**24.58**
		–	± 13.37	± 32.48	± 18.07	± 23.62	± 27.28	± 43.36	± 41.02	± 41.82

Source data used in Figure S5. Values presented represent mean ± SEM.

Table S5. High-glucose induced basal and chemoreflex-evoked tSNA responses with and without Exendin-4 in Wistar and SH rats

		**Glucose + Sham**				**Glucose + Exendin-4**			
		Control	2 min	10 min	20 min	Control	2 min	10 min	20 min
Glucose evoked tSNA: Change from baseline (%)	Wistar	**0.00**	**43.71**	**74.61**	**87.24**	**0.00**	**-23.54**	**-16.16**	**1.46**
		–	± 26.24	± 28.83	± 39.81	–	± 8.59	± 4.01	± 14.86
	SHR	**0.00**	**12.02**	**37.30**	**47.25**	**0.00**	**-36.01**	**-18.91**	**8.34**
		–	± 6.7	± 23.8	± 31.01	–	± 11.72	± 12.16	± 12.01
Resting tSNA: Change from baseline (%)	Wistar	**0.00**	**7.05**	**11.26**	**23.72**	**0.00**	**-6.83**	**-2.65**	**1.10**
		–	± 1.43	± 1.91	± 2.64	–	± 2.65	± 1.51	± 1.35
	SHR	**0.00**	**11.97**	**30.84**	**37.75**	**0.00**	**-6.71**	**0.15**	**8.39**
		–	± 2.79	± 7.16	± 8.14	–	± 2.03	± 2.52	± 3.20

Source data used in Figure 5. Values presented represent mean ± SEM.

Table S6. Full details of experimental animals used in the study

University of Bristol (UK)

ID	Sex	Age (weeks)	Date of collection	Weigth (g)	ID	Sex	Age (weeks)	Date of collection	Weigth (g)
**Spontaneously Hypertensive Rats (SHR/NHsd) Envigo**					**Wistar-Kyoto (WKY/NHsd) Envigo**				
**Transcriptomic RNA-seq analysis**									
SHR 1	♂	13	10/01/2019	294	WKY 1	♂	13	08/01/2019	268
SHR 2	♂	13	10/01/2019	271	WKY 2	♂	13	08/01/2019	250
SHR 3	♂	13	10/01/2019	261	WKY 3	♂	13	09/01/2019	261
SHR 4	♂	13	10/01/2019	276	WKY 4	♂	13	09/01/2019	253
SHR 5	♂	13	11/01/2019	281	WKY 5	♂	13	09/01/2019	260
SHR 6	♂	13	11/01/2019	295	WKY 6	♂	13	05/02/3019	257
**RT-qPCR analysis: male rats**									
SHR 1	♂	13	10/01/2019	274	WKY 1	♂	13	08/01/2019	248
SHR 2	♂	13	11/01/2019	282	WKY 2	♂	13	08/01/2019	249
SHR 3	♂	13	05/02/2019	310	WKY 3	♂	13	05/02/2019	251
SHR 4	♂	13	05/02/3019	320	WKY 4	♂	13	09/01/2019	272
SHR 5	♂	13	06/02/2019	315	WKY 5	♂	13	06/02/2019	262
SHR 6	♂	13	06/02/2019	320	WKY 6	♂	13	06/02/2019	264
**RT-qPCR analysis: dioestrus female rats**									
SHR 1	♀	13	31/07/2019	174	WKY 1	♀	12	17/09/2019	190
SHR 2	♀	13	31/07/3019	186	WKY 2	♀	12	18/09/2019	184
SHR 3	♀	13	31/07/2019	162	WKY 3	♀	12	18/09/2019	175
SHR 4	♀	13	01/08/2019	162	WKY 4	♀	12	20/09/2019	180
SHR 5	♀	13	01/08/2019	172	WKY 5	♀	12	20/09/2019	194
SHR 6	♀	13	02/08/3019	176	WKY 6	♀	13	26/09/2019	206
**RT-qPCR analysis: pre-hypertensive rats**									
SHR 1	♂	4	01/05/2019	54.5	WKY 1	♂	4	01/05/2019	51.3
SHR 2	♂	4	01/05/2019	52.0	WKY 2	♂	4	01/05/2019	51.7
SHR 3	♂	4	01/05/2019	49.6	WKY 3	♂	4	01/05/2019	49.1
**Immunohistochemistry**									
SHR 1	♂	12	17/10/2019	269	WKY 1	♂	12	17/10/2019	233
SHR 2	♂	12	17/10/2019	287	WKY 2	♂	12	17/10/2019	237
SHR 3	♂	12	18/10/2019	277	WKY 3	♂	12	18/10/2019	252
SHR 4	♂	12	15/11/2018	289	WKY 4	♂	12	15/11/2018	310
**LUXendin-645 experiment**									
SHR 1	♂	8	01/09/2020	271	WKY 1	♂	6	12/08/2020	139
SHR 2	♂	8	01/09/2020	249	WKY 2	♂	8	01/09/2020	250
**Wistar (Crl:WI; outbred) Charles Rivers**									
**RT-qPCR analysis: Wistar rats**									
Wistar 1	♂	9 - 10	12/08/2020	323					
Wistar 2	♂	9 - 10	12/08/2020	314					

Wistar 3	♂	9 - 10	12/08/2020	344
Wistar 4	♂	9 - 10	12/08/2020	347

University of Auckland (NZ)

ID	Sex	Age (weeks)	Date of collection	Weigth (g)	ID	Sex	Age (weeks)	Date of collection	Weigth (g)
**Spontaneously Hypertensive Rat**					**Wistar**				
**Chemoreflex: Exendin-4 and Exendin-3 in sequence**									
SHR 1	♂	5	18/06/2020	59	Wist 1	♂	4	29/05/2020	57
SHR 2	♂	5	18/06/2020	50	Wist 2	♂	5	04/06/2020	54
SHR 3	♂	5	19/06/2020	53	Wist 3	♂	5	04/06/2020	64
SHR 4	♂	4	24/06/2020	58	Wist 4	♂	5	10/06/2020	54
SHR 5	♂	4	25/06/2020	54	Wist 5	♂	5	10/06/2020	51
SHR 6	♂	5	02/07/2020	51	Wist 6	♂	5	11/06/2020	67
SHR 7	♂	4	03/07/2020	50	Wist 7	♂	4	11/06/2020	69
SHR 8	♂	4	03/07/2020	50	Wist 8	♂	4	17/06/2020	59
**Baseline Exendin-4**									
SHR 1	♂	4	31/08/2020	52	Wist 1	♂	4	01/09/2020	61
SHR 2	♂	5	02/09/2020	50	Wist 2	♂	5	02/09/2020	60
SHR 3	♂	4	08/09/2020	64	Wist 3	♂	5	04/09/2020	54
SHR 4	♂	5	01/10/2020	55	Wist 4	♂	4	04/09/2020	51
**Baseline Exendin-3**									
SHR 1	♂	5	01/10/2020	55	Wist 1	♂	4	08/09/2020	51
SHR 2	♂	5	05/10/2020	65	Wist 2	♂	5	28/09/2020	54
SHR 3	♂	5	07/10/2020	50	Wist 3	♂	4	29/09/2020	56
SHR 4	♂	4	07/10/2020	50	Wist 4	♂	4	29/09/2020	58
**Chemoreflex: Exendin-3**									
SHR 1	♂	4	08/10/2020	50	Wist 1	♂	4	13/10/2020	63
SHR 2	♂	5	09/10/2020	50	Wist 2	♂	5	13/10/2020	53
SHR 3	♂	5	16/10/2020	52	Wist 3	♂	5	16/10/2020	63
SHR 4	♂	5	27/10/2020	50	Wist 4	♂	5	16/10/2020	60
SHR 5	♂	5	30/10/2020	57	Wist 5	♂	4	27/10/2020	55
SHR 6	♂	4	09/11/2020	60	Wist 6	♂	5	27/10/2020	55
**Glucose study: hyperglycaemia only**									
SHR 1	♂	4	16/11/2020	61	Wist 1	♂	5	05/11/2020	54
SHR 2	♂	4	16/11/2020	60	Wist 2	♂	4	05/11/2020	58
SHR 3	♂	4	17/12/2020	50	Wist 3	♂	5	09/11/2020	59
SHR 4	♂	4	17/12/2020	51	Wist 4	♂	5	11/11/2020	57
**Glucose study: hyperglycaemia + Exendin-4**									
SHR 1	♂	4	18/12/2020	54	Wist 1	♂	5	14/12/2020	59
SHR 2	♂	5	18/12/2020	54	Wist 2	♂	5	14/12/2020	63
SHR 3	♂	4	25/01/2021	55	Wist 3	♂	5	16/12/2020	57
SHR 4	♂	4	25/01/2021	60	Wist 4	♂	4	16/12/2020	58
**Dose response to NaCN**									
					Wist 1	♂	5	Nov/2021	63
					Wist 2	♂	5	Nov/2021	67

	Wist 3	♂	4	Nov/2021	55
	Wist 4	♂	5	Nov/2021	63
	Wist 5	♂	4	Nov/2021	57
	Wist 6	♂	5	Nov/2021	62
	Wist 7	♂	5	Nov/2021	58
**Carotid sinus nerve activity Exendin-4**					
	Wist 1	♂	4	Nov/2021	54
	Wist 2	♂	4	Nov/2021	55
	Wist 3	♂	5	Nov/2021	62
	Wist 4	♂	5	Nov/2021	60
	Wist 5	♂	5	Nov/2021	62
	Wist 6	♂	5	Nov/2021	61

University of Belgrade (SRB)

ID	Sex	Weigth (g)	ID	Sex	Weigth (g)
**Spontaneously Hypertensive Rats**			**Wistar-Kyoto Rats**		
**Arterial BP recording**					
SHR 1	♂	333	WKY 1	♂	315
SHR 2	♂	315	WKY 2	♂	300
SHR 3	♂	300	WKY 3	♂	245
SHR 4	♂	294	WKY 4	♂	272
SHR 5	♂	302	WKY 5	♂	327
SHR 6	♂	311	WKY 6	♂	344
SHR 7	♂	294	WKY 7	♂	364
SHR 8	♂	317	WKY 8	♂	294
**Arterial blood collection**					
SHR 1	♂	314	WKY 1	♂	362
SHR 2	♂	294	WKY 2	♂	315
SHR 3	♂	303	WKY 3	♂	243
SHR 4	♂	294	WKY 4	♂	327
SHR 5	♂	332	WKY 5	♂	344
SHR 6	♂	283	WKY 6	♂	297

Table S7. RNA yields and integrity used for RNA-seq

Animal	Side	RIN Score	Qubit HS RNA assay (ng/µL)	Total RNA Yield (ng)
**Wistar-Kyoto (WKY/NHsd)**				
WKY 1	CB L	7.7	7.98	95.76
	CB R	7.5	8.7	104.4
WKY 2	CB L	7.4	10.3	123.6
	CB R	7	9.36	112.32
WKY 3	CB L	8.2	11.3	135.6
	CB R	6.9	12	144
WKY 4	CB L	8.2	10.8	129.6
	CB R	7.4	14.7	176.4
WKY 5	CB L	7.9	12.7	152.4
	CB R	7.4	11.6	139.2
WKY 6	CB L	7.4	6.68	80.16
	CB R	8.1	6.82	81.84
**Spontaneously Hypertensive Rat (SHR/NHsd)**				
SHR 1	CB L	7.3	15	180
	CB R	8.1	8	96
SHR 2	CB L	8	14.3	171.6
	CB R	7.7	18.9	226.8
SHR 3	CB L	7.1	12.8	153.6
	CB R	7.7	11.5	138
SHR 4	CB L	7.7	10.5	126
	*CB R	6.7	9.94	119.28
SHR 5	CB L	8.8	11.5	138
	CB R	8.7	12.6	151.2
SHR 6	CB L	7.4	14.9	178.8
	CB R	7.7	13.8	165.6

Sample 'SHR 4 right side' was removed from analysis after being identified as an outlier following initial PCA analysis (Figure S1c**)*

RNA integrity numbers and total RNA yields of individual samples used for RNA sequencing.

Table S8. PCR primers used in the study

Species	Target	Symbol	Forward 5'–3'	Reverse 5'–3'
Rat	ENSRNOG00000001152	*Glp1r*	ATCAAAGACGCTGCCCTCAA	CCACGCAGTATTGCATGAGC
Rat	ENSRNOG00000007647	*Oprk1*	AAACATCAGGGACGTGGACC	CTCCCTTCCCAAATCAGCGT
Rat	ENSRNOG00000054204	*Gria2*	GGGATTCACTGATGGGGACC	CATCATACGTCAGGGCCGAA
Rat	ENSRNOG00000006595	*Htr3a*	TGACTGCTCAGCCATGGGAAA	GTGGAAGAGGGCTATCTCTGC
Rat	ENSRNOG00000055382	*Hcn1*	GCACCGATACCAAGGCAAGA	ATTGGGATCCGCGTTAGCAA
Rat	ENSRNOG00000009450	*Hcn4*	ACGACTACTACGAACACCGC	GTCTGCATTGGCGAACAGTG
Rat	ENSRNOG00000029986	*Insr*	GCTACCTGGCCACTATCGAC	AACTGCCCATTGATGACGGT
Rat	ENSRNOG00000003241	*Gabrg2*	GAAAAACCCTGCCCCTACCA	TGCGAATGTGTATCCTCCCG
Rat	ENSRNOG00000007682	*Gria3*	CCATGCTCTTGTCAGCTTCG	TGTGCTCCTGAACCGTGTTT
Rat	ENSRNOG00000045816	*Gria1*	GGACAACTCAAGCGTCCAGA	CACAGTAGCCCTCATAGCGG
Rat	ENSRNOG00000013902	*P2ry12*	TTGCACGGATTCCCTACACC	GGGTGCTCTCCTTCACGTAG
Rat	ENSRNOG00000001296	*P2rx7*	TGTGCTTTCTTCGGCTACTCT	AGGCTCACTCTGTTTCGGC
Rat	ENSRNOG00000048145	*Sstr1*	ACGCGATGGTGCTGCTTATT	CTGGATACACTGGAGCGTGG
Rat	ENSRNOG00000004317	*Vipr2*	ATAGGCGCGAGACTGAGGAA	ATTCTGGGTGGATGCTGCTC
Rat	ENSRNOG00000014149	*Npy1r*	ACGGACGTGTCCAAGACTTC	CCCTGGGAGCACAGGTAATG
Rat	ENSRNOG00000018346	*Agtr1a*	TTCGTGGCTTGAGTCCTGTT	GGTGATCACTTTCTGGGAGGG
Rat	ENSRNOG00000017060	*Ryr2*	CAGAGGACCTGACTGTTCTCC	GGATTGTTCTTCGGTCTTGGC
Rat	ENSRNOG00000006641	*Dbh*	CTGGTGTACACGCCCTTGAT	AGGCAAAGATGCGGATTCCA
Rat	ENSRNOG00000013829	*Chrna3*	GGGGGACACTCCTAAGACGA	CAGCTTTTGGAGTCTGCACG
Rat	ENSRNOG00000017002	*Adrb1*	CTACAACGACCCCAAGTGCT	ACGTAGAAGGAGACGACGGA
Rat	ENSRNOG00000002793	*Sstr2*	TTGACCTCAACGGCTCACTG	CGTAGCGGAGGATGACGTAG
Rat	ENSRNOG00000050006	*Agtr2*	TGCTCTGACCTGGATGGGTA	AGCTGTTGGTGAATCCCAGG
Rat	ENSRNOG00000000522	*Cpne5*	TTTACTGTCTGGTCCTGTGGC	GCAAGGGTCAGAGCTGTTTTC
Rat	ENSRNOG00000015321	*Moxd1*	ACAACGCAGAGTGGTCGATT	CGCTCAGCATACTTCCGAGT
Rat	ENSRNOG00000000728	*Clic2*	CAAGCTTAGCGCTCAACACC	TTTCTCCGTCACTTCCAGCC
Rat	ENSRNOG00000018692	*Mc4r*	ACGGGTCAGAAACCATCGTC	GCGAGCAAGGAGCTACAGAT
Rat	ENSRNOG00000019163	*Syt6*	AGAGTTCGAGTCACTGCTCC	GACAAGTCTCCACGTCCAGC
Rat	ENSRNOG00000008939	*Nxph1*	CGCTCCCTGCTCTGTAAAGT	GGCACACGTGACCAAGTAGA
Human	ENST00000373256.5	*GLP1R*	ATCACAGTGGCGAGAGGAGA	TTGCAAGCCCCAGTTTCACT
Human	ENSG00000180176	*TH*	CGTGCTAAACCTGCTCTTCTC	TTCAAACGTCTCAAACACCTTCA
Human	ENSG00000111640	*GAPDH*	AATCCCATCACCATCTTCCA	TGGACTCCA CGACGTACTCA

Full list of primers used in RT-qPCR experiments reported in the study

Table S9. Top 10 Upregulated/Downregulated DEGs in SHR CBs based on IUPHAR/BPS targets

IUPHAR/BPS Target Class	Top 10 Upregulated DEGs Symbol	LFC	p value	p.adj	Top 10 Downregulated DEGs Symbol	LFC	p value	p.adj
gpcr	*Mc4r*	2.57	1.02E-41	3.60E-39	*Prokr1*	-0.71	2.43E-09	9.19E-08
gpcr	*Gpr61*	2.04	6.33E-12	3.43E-10	*Gipr*	-0.73	7.36E-07	1.77E-05
gpcr	*Lpar3*	1.95	8.19E-14	5.94E-12	*Adgrb2*	-0.90	2.30E-16	2.17E-14
gpcr	*Chrm1*	1.79	2.29E-08	7.31E-07	*Ptger3*	-0.94	1.06E-03	9.21E-03
gpcr	*Ptgfr*	1.23	4.57E-09	1.65E-07	*Gpr139*	-0.99	2.08E-23	3.35E-21
gpcr	*Agtr2*	1.16	1.04E-18	1.26E-16	*Gpr157*	-1.07	2.99E-06	6.20E-05
gpcr	*Sctr*	1.16	9.26E-12	4.88E-10	*Gpr6*	-1.25	2.19E-06	4.68E-05
gpcr	*Oprl1*	1.01	7.51E-05	1.02E-03	*Opn4*	-1.95	7.02E-11	3.34E-09
gpcr	*Adgrg5*	0.96	1.46E-04	1.78E-03	*Glp1r*	-4.21	1.78E-71	1.03E-68
gpcr	*Sstr2*	0.92	1.61E-22	2.42E-20	*Oprk1*	-4.49	3.83E-18	4.41E-16
lgic	*Grid1*	0.91	4.27E-04	4.36E-03	*Gria1*	-0.31	6.80E-04	6.41E-03
lgic	*Grin2a*	0.74	7.47E-03	4.27E-02	*Gabrg2*	-0.33	6.37E-03	3.78E-02
lgic	*Chrna4*	0.74	4.83E-03	3.06E-02	*Chrna5*	-0.45	2.73E-05	4.26E-04
lgic	*Chrna3*	0.60	3.15E-10	1.34E-08	*Grik2*	-0.45	8.01E-06	1.46E-04
lgic	*Chrna7*	0.23	5.78E-03	3.51E-02	*Gria3*	-0.55	1.72E-08	5.75E-07
lgic	–	–	–	–	*Grin2c*	-0.78	4.08E-06	8.16E-05
lgic	–	–	–	–	*Chrnb4*	-0.95	6.94E-12	3.74E-10
lgic	–	–	–	–	*Htr3a*	-1.02	5.72E-03	3.48E-02
lgic	–	–	–	–	*Grin2b*	-1.53	1.68E-03	1.33E-02
lgic	–	–	–	–	*Gria2*	-1.53	4.67E-78	3.53E-75
vgic	*Kcnh1*	1.08	5.01E-04	4.97E-03	*Kcnd3*	-0.85	1.16E-04	1.47E-03
vgic	*Kcnt2*	0.78	4.38E-07	1.10E-05	*Kcnn2*	-0.86	1.41E-03	1.16E-02
vgic	*Kcnq1*	0.67	3.01E-03	2.12E-02	*Hcn1*	-0.90	5.41E-17	5.44E-15
vgic	*Scn9a*	0.62	1.63E-07	4.48E-06	*Trpv3*	-1.00	3.03E-06	6.26E-05
vgic	*Kcnq3*	0.46	2.45E-03	1.80E-02	*Kcnk2*	-1.09	2.52E-03	1.84E-02
vgic	*Kcnd1*	0.42	1.41E-04	1.74E-03	*Kcnc4*	-1.10	1.77E-05	2.91E-04
vgic	*Cacna1g*	0.41	4.54E-06	8.90E-05	*Kcnj16*	-1.15	4.71E-16	4.16E-14
vgic	*Cacna1a*	0.30	2.37E-03	1.76E-02	*Kcnj10*	-1.22	3.63E-21	5.10E-19
vgic	*Kcnh2*	0.28	1.87E-03	1.46E-02	*Kcnj11*	-1.45	7.47E-18	8.33E-16
vgic	*Trpm4*	0.27	4.19E-03	2.74E-02	*Cacna1e*	-1.51	2.97E-06	6.18E-05
transporter	*Slc9a4*	2.00	4.05E-16	3.62E-14	*Abca8*	-1.02	6.17E-51	2.62E-48
transporter	*Slc11a1*	1.97	6.17E-30	1.47E-27	*Slc6a11*	-1.10	6.81E-03	3.98E-02
transporter	*Slc27a6*	1.90	7.12E-12	3.82E-10	*Slc25a27*	-1.10	4.07E-34	1.14E-31
transporter	*Slc35c2*	1.45	3.62E-138	1.05E-134	*Slc4a4*	-1.42	1.64E-11	8.32E-10
transporter	*Slc43a3*	0.95	1.19E-30	2.95E-28	*Slc5a7*	-1.43	1.86E-06	4.03E-05
transporter	*Slc17a9*	0.89	2.58E-07	6.81E-06	*Slc27a5*	-1.44	2.38E-03	1.77E-02
transporter	*Slc8a2*	0.80	1.03E-12	6.24E-11	*Slco1b2*	-1.61	6.66E-03	3.92E-02
transporter	*Slc6a4*	0.67	2.02E-08	6.57E-07	*Slc27a2*	-1.66	1.75E-03	1.38E-02
transporter	*Atp8b1*	0.64	1.08E-28	2.39E-26	*Slc6a1*	-1.84	9.98E-21	1.37E-18
transporter	*Slc16a6*	0.64	1.74E-04	2.06E-03	*Abcc8*	-2.00	2.02E-15	1.67E-13
cat receptor	*Il1rl2*	1.99	5.03E-08	1.54E-06	*Itga6*	-0.48	7.98E-03	4.50E-02
cat receptor	*Il18r1*	0.82	2.39E-12	1.37E-10	*Bmpr1a*	-0.51	4.54E-16	4.03E-14
cat receptor	*Ptpru*	0.82	2.53E-06	5.32E-05	*Il13ra2*	-0.55	2.56E-03	1.86E-02
cat receptor	*Ret*	0.71	1.00E-13	7.07E-12	*Il17re*	-0.60	1.99E-08	6.48E-07
cat receptor	*Flt3*	0.67	3.93E-05	5.81E-04	*Dhx58*	-0.63	1.73E-10	7.76E-09
cat receptor	*Epha7*	0.65	2.86E-03	2.03E-02	*Ddx58*	-0.63	5.71E-08	1.73E-06
cat receptor	*Itga5*	0.56	7.36E-13	4.65E-11	*Insr*	-0.80	7.98E-28	1.69E-25
cat receptor	*Cntfr*	0.52	5.31E-03	3.29E-02	*Il17rb*	-0.81	7.00E-06	1.30E-04
cat receptor	*Nlrx1*	0.48	2.29E-09	8.71E-08	*Itgb8*	-0.89	2.56E-03	1.86E-02
cat receptor	*Itga11*	0.48	2.48E-04	2.79E-03	*Ephb1*	-1.12	3.34E-03	2.28E-02
nhr	*Nr1i3*	0.28	5.22E-03	3.25E-02	*Rarb*	-0.36	2.43E-03	1.79E-02
nhr	*Esrra*	0.26	3.99E-04	4.11E-03	*Esrrg*	-0.49	4.89E-03	3.09E-02
nhr	*Rxrb*	0.26	3.21E-04	3.46E-03	*Nr5a2*	-1.14	2.92E-05	4.53E-04
nhr	*Nr1h2*	0.15	3.02E-03	2.12E-02	–	–	–	–
enzyme	*Ephx2*	2.71	8.95E-119	1.42E-115	*F10*	-1.20	2.57E-03	1.87E-02
enzyme	*Cpa3*	2.31	2.92E-06	6.08E-05	*Tssk3*	-1.22	6.82E-05	9.36E-04
enzyme	*Cma1*	2.09	2.30E-08	7.33E-07	*Plg*	-1.28	1.76E-04	2.09E-03
enzyme	*Ptges*	1.43	3.62E-05	5.42E-04	*Adamts17*	-1.39	1.79E-03	1.41E-02
enzyme	*Pla2g2d*	1.29	8.74E-05	1.15E-03	*Cyp1a2*	-1.47	3.26E-03	2.25E-02
enzyme	*Enpp2*	1.11	3.92E-68	2.20E-65	*Nek5*	-1.47	1.42E-06	3.17E-05
enzyme	*Camk4*	1.08	1.62E-10	7.27E-09	*Pon1*	-1.58	4.15E-03	2.72E-02
enzyme	*Cfd*	0.97	7.64E-04	7.07E-03	*Cyp39a1*	-1.62	4.54E-18	5.20E-16
enzyme	*Srms*	0.94	4.94E-12	2.69E-10	*Sgpp2*	-1.69	2.58E-12	1.46E-10
enzyme	*Ca9*	0.93	8.86E-04	7.99E-03	*Cth*	-1.83	2.36E-03	1.76E-02

Tabular summary of Figure 1e

Table S10. Bulk RNA-seq validation by qRT-PCR

	bulk RNA-seq			qRT-PCR		
**Gene symbol**	Replicates: n=6<br>Normalisation: DEseq2<br>Test: DESeq2 model<br>*Post hoc* correction:<br>Benjamin-Hodgberg			Replicates: n=6<br>Normalisation: delta Ct<br>Test: Mann-Whitney U test<br>*Post hoc* correction:<br>Benjamin-Hodgberg		
	**LFC**	***p* value**	**LFC**	**LFC**	***p* value**	***p* adj**
Oprk1	-4.49	3.83E-18	4.41E-16	-3.08	2.17E-03	6.06E-02
Glp1r	-4.21	1.78E-71	1.03E-68	-4.86	2.17E-03	3.03E-02
Gria2	-1.53	4.67E-78	3.53E-75	-1.16	2.60E-02	5.19E-02
Htr3a	-1.02	5.72E-03	3.48E-02	-0.98	1.32E-01	1.85E-01
Hcn1	-0.90	5.41E-17	5.44E-15	-0.87	2.17E-03	2.02E-02
Hcn4	-0.77	1.44E-05	2.46E-04	-0.96	4.33E-03	1.10E-02
Insr	-0.80	7.98E-28	1.69E-25	-0.50	2.17E-03	1.52E-02
Gabrg2	-0.33	6.37E-03	3.78E-02	-0.94	1.52E-02	3.26E-02
Gria3	-0.55	1.72E-08	5.75E-07	-0.40	9.31E-02	1.37E-01
Gria1	-0.31	6.80E-04	6.41E-03	-0.48	8.66E-03	2.02E-02
P2rx7	-0.26	7.88E-03	4.45E-02	0.34	3.10E-01	3.61E-01
P2ry12	-0.23	6.72E-03	3.94E-02	0.05	8.18E-01	8.49E-01
Agtr1a	0.25	7.92E-03	4.47E-02	0.68	4.11E-02	6.77E-02
Ryr2	0.28	1.13E-02	5.79E-02	0.72	4.11E-02	6.40E-02
Npy1r	0.39	4.44E-06	8.72E-05	0.46	3.10E-01	3.47E-01
Sstr1	0.40	2.92E-08	9.24E-07	0.19	2.40E-01	2.93E-01
Chrna3	0.60	3.15E-10	1.34E-08	0.86	1.80E-01	2.40E-01
Dbh	0.71	1.71E-07	4.69E-06	0.33	6.99E-01	7.53E-01
Vipr2	0.73	8.74E-05	1.15E-03	0.03	1.00E+00	1.00E+00
Adrb1	0.75	3.58E-06	7.28E-05	0.89	2.60E-02	4.85E-02
Sstr2	0.92	1.61E-22	2.42E-20	1.16	2.60E-02	4.54E-02
Agtr2	1.16	1.04E-18	1.26E-16	1.40	2.17E-03	1.21E-02
Moxd1	1.51	1.58E-13	1.10E-11	1.77	2.17E-03	1.01E-02
Cpne5	2.15	6.35E-60	3.25E-57	0.67	1.91E-01	2.42E-01
Clic2	2.27	1.12E-103	1.50E-100	2.52	2.17E-03	8.66E-03
Syt6	2.52	8.33E-54	3.72E-51	2.61	2.17E-03	7.58E-03
Mc4r	2.57	1.02E-41	3.60E-39	2.34	2.17E-03	6.74E-03
Nxph1	3.01	2.47E-30	6.05E-28	3.42	2.17E-03	6.06E-03

Tabular summary of **Figure 1f**

Data sets

Data set 1. Full list of differentially expressed genes in SH rat carotid bodies

Full list of differentially expressed genes between carotid bodies of SH and WKY rats identified by RNA-seq and DEseq2 analysis.

Data set 2. Full list of pathway enrichment analysis results

Full list of enrichment analysis results against Gene Ontology (MF, BP, CC), KEGG pathways, REACTOME pathways and Wiki Pathways databases. Key: GSEA – gene set enrichment analysis.
