# Supplementary material for: GLP1R Attenuates Sympathetic Response to High Glucose via Carotid Body Inhibition
Source: Circ Res. 2022 Feb 1;130(5):694–707. doi: 10.1161/CIRCRESAHA.121.319874 (PMC8893134; doi:10.1161/CIRCRESAHA.121.319874)
Supplement: Supplementary file 3 [file res-130-694-s003.pdf]

Major Resources Table

In order to allow validation and replication of experiments, all essential research materials listed in the Methods should be included in the Major Resources Table below. Authors are encouraged to use public repositories for protocols, data, code, and other materials and provide persistent identifiers and/or links to repositories when available. Authors may add or delete rows as needed.

Animals (in vivo studies)

Species	Vendor or Source	Background Strain	Sex	Persistent ID / URL
Rattus norvegicus	Envigo, UK	Spontaneously hypertensive inbred rats (**SHR/NHsd)**	Male/ Female	https://www.envigo.com/model/shr-nhsd?selctry=U.K.&ctry=
Rattus norvegicus	Envigo, UK	Wistar Kyoto inbred rats **WKY/NHsd**	Male/ Female	https://www.envigo.com/model/wky-nhsd
Rattus norvegicus	Charles Rivers, UK	Wistar IGS Rat Outbred (**Crl:WI**)	Male	https://www.criver.com/products-services/find-model/wistar-igs-rat?region=3671

Genetically Modified Animals

	Species	Vendor or Source	Background Strain	Other Information	Persistent ID / URL
**Parent - Male**					
**Parent - Female**					

Antibodies

Target antigen	Vendor or Source	Catalog #	Working concentration	Lot # (preferred but not required)	Persistent ID / URL
GLP1R mouse, rat;	Abcam	ab218532	IHC: 1:500 WB: 1:1000		http://antibodyregistry.org/AB_2864762
Glucagon-like peptide 1 receptor epitope: aa 24-145; 24467746 human, primate	DSHB	MAB3F52	ICC: 1:30		http://antibodyregistry.org/AB_2618100
Tyrosine Hydroxylase (TH, Tyrosine Monooxygenase) chicken/avian, fish, human, mouse, other, rat, simian, xenopus, chicken, human, mouse, monkey	Millipore	MAB318	IHC: 1:500		http://antibodyregistry.org/AB_2201528

DOI [to be added]

Tyrosine Hydroxylase antibody - Neuronal Marker mouse, rat, human, mouse, rat	Abcam	ab113	IHC: 1:500		http://antibodyregistry.org/AB_297905
mouse anti-GLP-1(7-36) amide (NBP2-23558, Novus Biologicals); reactivity human, mouse, mammal	Novus-bio	NBP2-23558	IHC: 1:200		https://www.novusbio.com/products/glp1-antibody-4_nbp2-23558
PGP9.5 antibody [13C4 / I3C4] - Neuronal Marker sheep, mouse, rat, canine, guinea pig, human, porcine, rabbit, human, mouse, rat, dog, guinea pig, pig, rabbit, sheep	Abcam	ab8189	IHC: 1:1000		http://antibodyregistry.org/AB_306343
Donkey Anti-Mouse IgG (H+L) Antibody, Alexa Fluor 488 Conjugated	Molecular Probes	A21202	IHC: 1:500		http://antibodyregistry.org/AB_141607
Goat anti-Mouse IgG (H+L) Highly Cross-Adsorbed Secondary Antibody, Alexa Fluor 568	Molecular Probes	A11031	IHC: 1:500		http://antibodyregistry.org/AB_144696
Anti-Rabbit IgG (H+L), highly cross-adsorbed, CF™ 633 antibody produced in donkey	Sigma-Aldrich	SAB4600132	IHC: 1:500		https://www.sigmaaldrich.com/GB/en/product/sigma/sab4600132
Donkey anti-Rabbit IgG (H+L) Highly Cross-Adsorbed Secondary Antibody, Alexa Fluor 568	Molecular Probes	A10042	IHC: 1:500		http://antibodyregistry.org/AB_2534017
Donkey Anti-Rabbit IgG (H+L) Antibody, Alexa Fluor 488 Conjugated	Molecular Probes	A21206	IHC: 1:500		http://antibodyregistry.org/AB_2535792
Anti-Mouse IgG (whole molecule)-	Sigma -	A9044	WB: 1:10000		http://antibodyregistry.org/AB_258431

DOI [to be added]

Peroxidase antibody produced in rabbit	Aldrich				
Anti-Rabbit IgG (whole molecule)-Peroxidase antibody produced in goat	Sigma-Aldrich	A0545	WB: 1:10000		http://antibodyregistry.org/AB_257896
Donkey Anti-Rabbit IgG Polyclonal antibody, Cyanine 3 Conjugated	Millipore	AP182C	IHC: 1:400		http://antibodyregistry.org/AB_92588
Donkey Anti-Sheep IgG (H+L) Antibody, Alexa Fluor 488 Conjugated	Molecular Probes	A11015	IHC: 1:300		http://antibodyregistry.org/AB_141362
Donkey Anti-Mouse IgG Polyclonal antibody, Cyanine 5 Conjugated	Millipore	AP192S	IHC: 1:400		http://antibodyregistry.org/AB_92656

DNA/cDNA Clones

Clone Name	Sequence	Source / Repository	Persistent ID / URL

Cultured Cells

Name	Vendor or Source	Sex (F, M, or unknown)	Persistent ID / URL
Human Embryonic Kidney 293AAV Cell Line (HEK293AAV) immortalized cell line **Category:** Transformed cell line	Cell Biolabs	Female	RRID: CVCL_KA64
Rat adrenal medulla pheochromocytoma PC12 immortalized cell line **Category:** Cancer cell line	Prof Shelley J Allen (University of Bristol)	Male	RRID:CVCL_0481 https://web.expasy.org/cellosaurus/CVCL_0481
Chinese hamster ovary -K1 cell line stably expressing the human SNAP_GLP1R (Cisbio) (CHO-K1-SNAP_GLP1R)	Dr Ben Jones, Imperial College London	Female	https://www.nature.com/articles/s41467-020-14309-w#Sec14

DOI [to be added]

immortalized cell line **Category:** Spontaneously immortalized cell line			

Data & Code Availability

Description	Source / Repository	Persistent ID / URL
RNA-STAR	**Github**	**RRID:SCR_004463** http://code.google.com/p/rna-star/
featureCounts	Sourceforge	**RRID:SCR_012919** http://bioinf.wehi.edu.au/featureCounts/ http://subread.sourceforge.net/
DEseq2	BioConductor	**RRID:SCR_015687** https://bioconductor.org/packages/release/bioc/html/DESeq2.html
ClusterProfiler	BioConductor	**RRID:SCR_016884** **http://bioconductor.org/packages/release/bioc/html/clusterProfiler.html**
GAGE	BioConductor	**RRID:SCR_017067** **http://bioconductor.org/packages/gage/**
Pathview	BioConductor	**RRID:SCR_002732** **http://www.bioconductor.org/packages/release/bioc/html/pathview.html**

Other

Description	Source / Repository	Persistent ID / URL
DyLight 488 Lycopersicon Esculentum (Tomato) Lectin	Vector Laboratories	http://antibodyregistry.org/AB_2336404 https://vectorlabs.com/dylight-488-labeled-lycopersicon-esculentum-tomato-lectin-lel-tl.html
Exendin-4; Cat. No. 1933	Tocris	https://www.tocris.com/products/exendin-4_1933
Exendin-3; Cat. No. 2081	Tocris	https://www.tocris.com/products/exendin-3-9-39-amide_2081
LUXendin-645, a far-red fluorescent GLP1R antagonistic peptide label	Professor David J. Hodson (University of Birmingham), and Dr Johannes Broichhagen (Leibniz-Forschungsinstitut für Molekulare Pharmakologie)	https://www.nature.com/articles/s41467-020-14309-w#Abs1
SNAP-tag substrate label (SBG-TMR)	Professor David J. Hodson (University of Birmingham), and	https://pubs.rsc.org/en/content/articlehtml/2020/sc/d0sc02794d

DOI [to be added]

	Dr Johannes Broichhagen (Leibniz-Forschungsinstitut für Molekulare Pharmakologie)	

DOI [to be added]
