# Supplementary material for: GLP1R Attenuates Sympathetic Response to High Glucose via Carotid Body Inhibition
Source: Circ Res. 2022 Feb 1;130(5):694–707. doi: 10.1161/CIRCRESAHA.121.319874 (PMC8893134; doi:10.1161/CIRCRESAHA.121.319874)

Full unedited gel for **Supp Figure 3b**

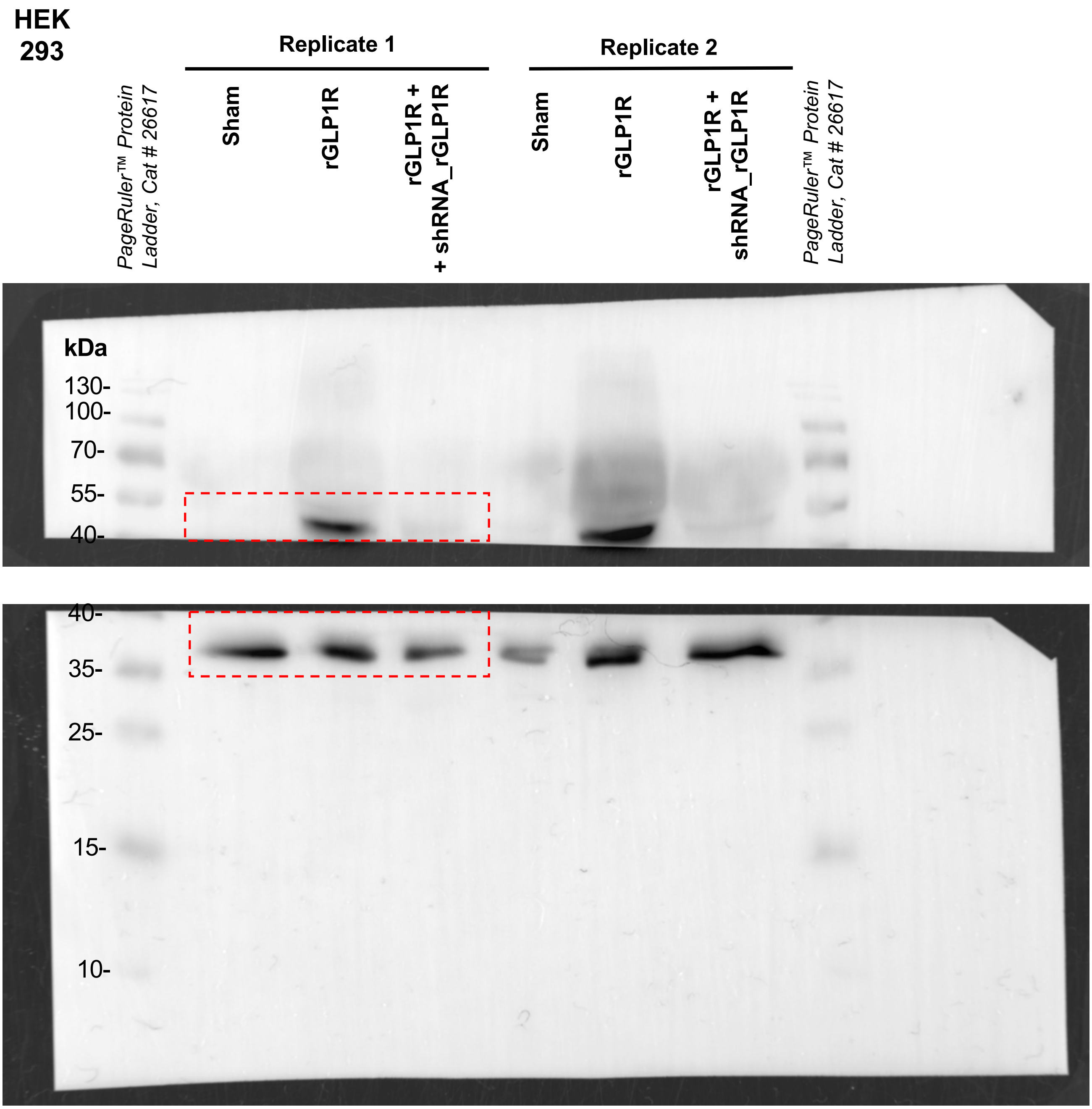

Following SDS-PAGE and protein transfer membrane was cut at 40 kDa protein ladder mark

- - - - lanes corresponding to those shown cropped in the **Supp Figure 3b**

Supp Figure 3b

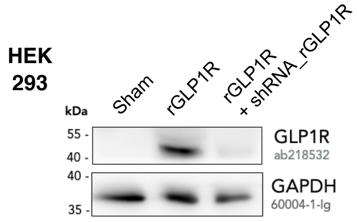

Supplement: Supplementary file 5 [file res-130-694-s005.pdf]
